# Integrated Multi-Omics and Network Pharmacology Reveal the Targeted Alleviation of MASLD by Litchi Pericarp Oligomeric Polyphenols via Quercetin

**DOI:** 10.3390/antiox15101246

**Published:** 2026-09-27

**Authors:** Lu Xiao, Jin-Wen Tang, Ya Mao, Yuan-Shan Yu, Xin Li, Wen-Yong Lou

**Affiliations:** 1 Lab of Applied Biocatalysis, School of Food Science and Engineering, South China University of Technology, No. 381 Wushan Road, Guangzhou 510640, China; xl2691915147@163.com (L.X.); fetang9@mail.scut.edu.cn (J.-W.T.); miaya1999@163.com (Y.M.); 2 Sericultural & Agri-Food Research Institute, Guangdong Academy of Agricultural Sciences, No. 133 Yiheng Street, Dongguanzhuang Road, Tianhe District, Guangzhou 510610, China; yuyuanshan@gdaas.cn

**Keywords:** MASLD, *Litchi chinensis*, pericarp oligomeric polyphenols, quercetin, multi-omics, network pharmacology

## Abstract

Metabolic dysfunction-associated steatotic liver disease (MASLD) represents a major global metabolic health burden, driving an urgent demand for safe, natural bioactive therapeutics derived from sustainable botanical sources. This study aimed to investigate the therapeutic efficacy and multi-target molecular mechanisms of litchi pericarp oligomeric polyphenols (LPPO) from *Litchi chinensis* Sonn. cv. Feizixiao against MASLD. A comprehensive multi-tiered pharmacological study design was conducted, integrating computational target prediction (network pharmacology and molecular docking) with in vitro cellular and in vivo animal disease models, combined with multi-omics profiling. Palmitate/oleate (FFA)-induced HepG2 cells and high-fat-diet (HFD)-fed C57BL/6J mice (JAX substrain) were treated with LPPO to evaluate hepatic steatosis, serum lipid profiles, oxidative stress parameters, and fecal lipid excretion. Integrated transcriptomics, metabolomics, network pharmacology, and molecular docking were conducted to pinpoint core bioactive constituents and validate key molecular targets and signaling pathways. LPPO markedly attenuated hepatic lipid accumulation, normalized serum lipid levels (TC, TG, and LDL-C), restored cellular redox balance (SOD and MDA), and promoted systemic lipid clearance via enhanced fecal excretion both in vitro and in vivo. Multi-omics integration identified quercetin as the principal bioactive metabolite driving LPPO-mediated metabolic reprogramming. Mechanistically, LPPO directly bound ALOX5 and downregulated ALOX12B to inhibit arachidonic acid-mediated inflammatory cascades, while suppressing NOS2/DUOX1 and upregulating MPV17L2 to alleviate oxidative/nitrosative stress. Additionally, quercetin downregulated the CD274 (PD-L1) checkpoint axis to reverse hepatic immunosuppression, alongside modulating GMPR, BCKDHA, and IDI1 to rebalance cellular energy allocation and lipid remodeling. LPPO exerts potent multi-target therapeutic effects against MASLD by alleviating lipotoxicity, oxidative stress, and inflammatory cascades. These findings elucidate the molecular basis of LPPO and highlight the potential of litchi pericarp waste as a high-value phytomedicinal resource.

## 1. Introduction

Metabolic dysfunction-associated steatotic liver disease (MASLD), recently redefined from the former non-alcoholic fatty liver disease (NAFLD) to emphasize the role of systemic metabolic impairment, has emerged as the most prevalent chronic liver disorder globally [1]. Current epidemiological evidence (based on data from 2022) indicates that MASLD affects approximately 32.4% of the global adult population, with a significantly higher rate among men (39.7%) than among women (25.6%), posing a formidable challenge to public health systems [2]. Given the high incidence of MASLD, it is essential to conduct research aimed at improving the management of this disorder. The pathogenesis of MASLD is intricately governed by the “multiple-hit” hypothesis, in which ectopic lipid accumulation and oxidative stress serve as the primary driving forces. Within this framework, excessive intracellular lipid accumulation leads to lipotoxicity, which subsequently triggers a cascade of mitochondrial dysfunction, inflammatory responses, and progressive cellular damage [3,4,5,6]. Contemporary strategies against MASLD primarily rely on lifestyle modifications, such as dietary interventions and weight loss. These are often combined with pharmacological treatments, including insulin sensitizers, GLP-1 receptor agonists, and recently approved THR-β agonists [7,8]. However, the long-term use of current MASLD therapies is frequently limited by varied patient responsiveness, contraindications, and potential adverse side effects, including gastrointestinal and cardiovascular risks. These limitations justify the critical importance of exploring new chemical entities and natural compounds. Investigating plant-derived bioactives like LPPO provides value far beyond addressing disease prevalence; it offers insights into alternative, multi-targeted therapeutic modalities with potentially superior safety profiles.

Litchi (*Litchi chinensis* Sonn.) is a commercially significant subtropical fruit widely cultivated for its unique flavor and nutritional profile. However, its industrial processing generates vast quantities of pericarp waste, accounting for 15% of the total fruit mass [9]. Meanwhile, the litchi pericarp is an important source of bioactive phytochemicals. In fact, proanthocyanidin A2, proanthocyanidin B2, epicatechin, rutin and catechin are the main polyphenolic compounds in litchi pericarp extract [10,11,12]. About 60% of the polyphenols are proanthocyanidins, which exist in the form of oligomers. Therefore, it is necessary to study litchi pericarp oligomeric polyphenols (LPPO). Despite the well-documented antioxidant and anti-inflammatory potencies of LPPO, a critical knowledge gap remains regarding the variability of phenolic profiles across different litchi cultivars [13,14,15]. Previous studies have largely overlooked how these varietal differences influence the biological efficacy of the extracts, a factor that is essential for the systematic and high-value valorization of litchi waste as a functional food ingredient [16].

Polyphenols coordinate lipid metabolism by modulating the AMPK/PPARα/SREBP-1c axis and bolster cellular defenses through Nrf2/NF-κB signaling. They also preserve mitochondrial integrity and reduce ROS mediated by the SIRT1-PGC-1α signaling pathway [17,18,19,20,21]. Studies have shown that polyphenols from different sources exhibit distinct alleviating effects and mechanisms of action. Blueberry leaf polyphenols mitigate non-alcoholic fatty liver disease by improving mitochondrial function and oxidative defense [22], whereas pomegranate polyphenols reduce inflammation and liver injury through the NF-κB and Nrf2/GSH axes [23]. However, most studies focus on phenotypic changes or gene expression levels (transcriptomics) without addressing the direct molecular interactions between specific monomers and their protein targets. The absence of evidence for direct ligand–receptor binding leaves a gap in our understanding of the precise molecular mechanisms. Integrating transcriptomics, network pharmacology, and molecular docking techniques to simulate physical binding processes can provide a robust strategy for bridging this gap.

Therefore, in the current study, we integrated metabolomics, transcriptomics, network pharmacology, and molecular docking strategies to systematically investigate the hypolipidemic effects and mechanism of LPPO. We first selected litchi pericarp procyanidin oligomers (LPPO) from the Feizixiao cultivar and validated their efficacy in FFA-induced HepG2 cells and C57BL/6J mice (JAX substrain). Subsequently, transcriptomics and network pharmacology were integrated to explore key signaling pathways and potential targets. Finally, the underlying mechanisms were elucidated by simulating direct interactions between LPPO monomers and core targets, via molecular docking, which was further verified by Western blot analysis. This research provides a theoretical foundation for the high-value utilization of litchi pericarp as a functional food ingredient.

## 2. Materials and Methods

### 2.1. LPPO Extraction and Detection

Fresh Feizixiao litchi fruits were purchased from Haoxin Jiali Foodstuffs Co., Ltd. (Maoming City, Guangdong Province, China) on 20 May 2024. The fruits were stored at −20 °C for exactly three weeks until use. While deep freezing preserves most bioactive compounds, prolonged storage at this temperature might induce minor modifications in polyphenol stability and oligomerization states, which was accounted for in our metabolomic profiling. LPPO was extracted using an ultrasound-assisted aqueous ethanol method (70% ethanol, 40 °C, 30 min) and subsequently purified via AB-8 macroporous resin. The metabolomic profiles were analyzed using UPLC-MS/MS to identify variety-specific markers [12,24].

The chromatographic conditions were as follows: ACQUITY UPLC HSS T3 column (100 Å, 1.8 µm, 2.1 mm × 100 mm) was used with a flow rate of 0.4 mL/min, column temperature of 40 °C, automatic sampler at 8 °C, and injection volume of 2 μL. Mobile phase A consisted of 0.1% formic acid in water; mobile phase B was acetonitrile containing 0.1% formic acid. The elution gradient was as follows: 0 min—5% mobile phase B; 1 min—5% mobile phase B; 4.7 min—95% mobile phase B; 6 min—95% mobile phase B; 6.1 min—5% mobile phase B; 8.5 min—5% mobile phase B. The mass spectrometry procedure was as follows: Data acquisition for DDA mass spectra in both positive and negative ion modes was performed using the Thermo Orbitrap Exploris 120 mass spectrometer under control of the Xcalibur software (version 4.7, Thermo). The HESI source was employed with a spray voltage of 3.5 kV/−3.0 kV, a sheath gas flow rate of 40 arb, an auxiliary gas flow rate of 10 arb, a capillary temperature of 320 °C, and an auxiliary gas temperature of 300 °C. Primary resolution was set to 60,000, with a scanning range of 70–1000 *m*/*z*. AGC settings were Target Standard with a Max IT of 100 ms; the top four most abundant ions were selected for secondary fragmentation with a dynamic exclusion time of 4 s and secondary resolution of 15,000. For HCD analysis, the collision energy was set to 30%, with AGC also configured as Target Standard and Max IT set to Auto.

Metabolite identification was performed using the Pearson PSNGM (Personal Bio Next-Generation Metabolomics Database), which includes self-built reference libraries, the mzCloud database (https://www.mzcloud.org/, accessed on 4 August 2026), LIPID MAPS (https://www.lipidmaps.org/, accessed on 4 August 2026), HMDB (https://hmdb.ca/, accessed on 4 August 2026), MoNA (https://mona.fiehnlab.ucdavis.edu/, accessed on 4 August 2026), NIST_2020_MSMS, and an AI-powered MS/MS spectrum prediction library. The primary search parameters were as follows: MS1 identification tolerance 0.01, MS2 identification tolerance 0.05, Smoothing level 3, Minimum peak height 10,000, Minimum peak width 5, Mass slice width 0.05, and Identification score cutoff 70 [25].

The total phenolic content (TPC) of the purified LPPO powder was determined using the standard Folin–Ciocalteu assay. Briefly, 20 µL of the appropriately diluted LPPO sample or standard was mixed with 100 µL of 10% (*v*/*v*) Folin–Ciocalteu reagent in a 96-well microplate. After a 5-min incubation in the dark, 80 µL of 7.5% (*w*/*v*) Na_2_CO_3_ solution was added to neutralize the reaction. The mixture was incubated at room temperature for 60 min in the dark, and the absorbance was measured at 765 nm using a microplate reader. Gallic acid (10–100 µg/mL) was used to construct the calibration curve. Based on the measurement results, the total polyphenol concentration of LPPO was determined to be 775.2 mg GAE/g.

To quantitatively determine the total quercetin concentration, accurately weigh 1.0 g of LPPO, add 20 mL of an 80% methanol solution containing 1% ascorbic acid, and subject the mixture to ultrasonic extraction at room temperature for 30 min. Then centrifuge at 8000× *g* for 10 min and combine the supernatants from both extractions. Take 10 mL of the extract, add hydrochloric acid to achieve a final concentration of 1.2 mol/L, seal the mixture under a nitrogen atmosphere, and hydrolyze it in an aqueous bath at 85 °C for 2 h; the reaction is terminated by placing the mixture in an ice bath. The hydrolyzed solution is diluted with methanol to a final volume of 25 mL and filtered through a 0.22 μm organic membrane filter. Analysis is performed using an HPLC-DAD system at a wavelength of 370 nm; the chromatographic column is a C18 reversed-phase column (4.6 × 250 mm, 5 μm); the mobile phase consists of a 0.1% formic acid aqueous solution and methanol at a flow rate of 1.0 mL/min and a column temperature of 30 °C. The total quercetin content is determined by means of a standard curve; the result is expressed as mg/g DW.

### 2.2. In Vitro Experiment

HepG2 cells were purchased from Procell Life Science & Technology Co., Ltd. (Wuhan, China; Cat. No. CL-0103), which corresponds to the Cellosaurus accession number CVCL_0027 and ATCC accession number HB-8065. Reagents for cell culture, including DMEM medium (Cat. No. 11995065), fetal bovine serum(Cat. No. 10099141), penicillin (Cat. No. 15140122), and streptomycin sulfate, were purchased from Thermo-Fisher Scientific. Cells were cultured in DMEM medium with 10% fetal bovine serum (FBS), 100 U/mL penicillin, and 100 μg/mL streptomycin sulfate in a humidified atmosphere of 5% CO_2_ at 37 °C. Cells were seeded into culture plates and treated when the confluence reached 70–80%. To induce the in vitro MASLD model, a free fatty acid (FFA) stock solution was first prepared by conjugating fatty acids with bovine serum albumin (BSA). Briefly, a 100 mM FFA mixture containing sodium oleate and sodium palmitate (2:1 molar ratio) was slowly added dropwise into a 50% serum-free BSA solution in DMEM under continuous vortexing. The mixture was then incubated at 37 °C for 30 min, with vortexing every 10 min, until the solution became completely clear. Following filter sterilization, the FFA-BSA stock solution was aliquoted into sterile brown centrifuge tubes and stored at −20 °C. Prior to treatment, the stored FFA stock was thawed and diluted with culture medium to a final working concentration of 1 mM. To detect the effects of LPPO on FFA-induced hepatic steatosis, HepG2 cells were co-treated with 1 mM FFA and varying doses of LPPO (0–250 ng/mL) for 24 h [22]. Cells cultured in DMEM with 10% FBS, 100 U/mL penicillin, and 100 μg/mL were used as the normal group [26].

Cell viability was evaluated using a Cell Counting Kit-8 (CCK-8; Beyotime, Shanghai, China, Cat. No. C0038) according to the manufacturer’s instructions [27]. The oil red O staining was performed using the Oil Red O Staining Kit (Beijing Solabao Technology Co., Ltd., Beijing, China, Cat. No. G1262) according to the manufacturer’s instructions. After the cells were lysed using BS205 NP-40 (Biosharp, Hefei, China), superoxide dismutase (SOD) levels were measured using a kit (Beyotime, Shanghai, China, Cat. No. S0101S), while triglyceride (TG, Nanjing Jiancheng, Nanjing, China, Cat. No. A110-1-1), total cholesterol (TC, Nanjing Jiancheng, Nanjing, China, Cat. No. A111-1-1), and malondialdehyde (MDA, Nanjing Jiancheng, Nanjing, China, Cat. No. A003-1-2) concentrations were determined with another kit according to the manufacturer’s instructions.

### 2.3. In Vivo Experiments

Nine male C57BL/6J mice (JAX substrain), aged 6 weeks, were purchased from Zhuhai Baishitong Biotechnology Co., Ltd., Zhuhai, China (Production License No. SCXK (Yue) 2025-0051). The animal experiment was conducted at Guangzhou Huateng Biomedical Technology Co., Ltd., Guangzhou, China (Animal Facility License No. SYXK (Yue) 2024-0307). The male C57BL/6J substrain was specifically selected because it is highly susceptible to diet-induced obesity and hepatic steatosis, while avoiding the hepatoprotective effects of estrogen present in female mice. All animal procedures were conducted in accordance with the ARRIVE guidelines and relevant institutional guidelines for the care and use of laboratory animals. The experimental protocol was reviewed and approved by the Laboratory Animal Use and Management Committee of Guangzhou Huateng Biomedical Technology Co., Ltd. (Approval No. B202511-17; approval date: 19 November 2025). This in vivo experiment was specifically designed as a mechanistic proof-of-concept study, which justifies the small sample size (3 mice per group) and the use of a single sex. After 1 week of acclimatization, the mice were randomly assigned to three groups based on body weight: model control group (MC, *n* = 3), low-dose LPPO group (LD, *n* = 3), and high-dose LPPO group (HD, *n* = 3). All mice were fed a high-fat diet (60% kcal from fat; D12492, Guangdong Provincial Medical Experimental Animal Center) for 8 weeks to establish the MASLD model. Although the 60% HFD does not perfectly replicate a realistic human Western diet, it was utilized as a robust and accelerated mechanistic model to rapidly induce severe lipotoxicity and ectopic lipid deposition, thereby providing a sufficiently rigorous metabolic challenge to evaluate the hepatoprotective efficacy of LPPO.

During the intervention period, the LD and HD groups received LPPO at 100 and 200 mg/kg/day, respectively, by oral gavage dissolved in purified water [22]. The MC group received an equal volume of vehicle. Mice were housed under controlled conditions with free access to food and water. Body weight, food intake, and water intake were monitored during the experiment per week. At the end of the intervention, mice were fasted for 12 h. Anesthesia was induced via intraperitoneal injection of a 1:1 (*v*/*v*) mixture of 0.5% Sumianxin II and 1% Zoletil 50 at a dose of 5 mL/kg. Blood was collected, and the mice were subsequently euthanized via exsanguination under the same deep anesthesia. Serum was separated for biochemical analysis. Liver tissues were collected immediately; part of the tissue was fixed in 4% paraformaldehyde for histological analysis, and the remaining tissue was stored at −80 °C for further biochemical assays.

The HE staining steps are as follows: Liver tissues were fixed in 4% paraformaldehyde (PFA) for 24 h, then flushed with tap water for 6 h. The tissues were dehydrated through a series of alcohols with gradient concentrations and xylene. Specifically, sections were stained with hematoxylin for 10 min, differentiated in 0.5% acid alcohol for 2 s, blued in running water, and counterstained with a 0.5% aqueous eosin solution for 6 min. Finally, the slides were dehydrated through a gradient ethanol series, cleared in xylene, and mounted with neutral balsam. The sealed sections were observed under a microscope (Nikon ECLIPSE 50i, Tokyo, Japan). To quantify hepatic steatosis from the H&E sections, the standard NAFLD/MASLD activity score (NAS) was applied to systematically evaluate steatosis, lobular inflammation, and hepatocellular ballooning.

The steps for oil red O staining are as follows. The frozen liver was sliced and allowed to dry and warm at room temperature. The slices were then fixed in 4% polyformaldehyde for 10 min and washed with distilled water. To prevent moisture from affecting subsequent staining, the slices were rapidly rinsed in 60% isopropanol for 15–20 s, and then incubated in freshly prepared, filtered Oil Red O solution under light protection for 10–15 min to stain lipid droplets. After staining, the slices were briefly washed in 60% isopropanol for 15–20 s to remove background coloration, then immediately rinsed with distilled water. Next, the nuclei were counterstained with hematoxylin for 1–3 min, rinsed under running water to restore the blue coloration, and finally covered with glycerol-gelatin. The sealed sections were observed under a microscope (Nikon ECLIPSE 50i, Tokyo, Japan).

Serum levels of TC, TG, HDL-C, LDL-C, and TBA were measured using the BS-240VET veterinary fully automated biochemical analyzer (Shenzhen Mindray Animal Medical Technology Co., Ltd., Shenzhen, China). SOD content in serum and liver was measured using a kit (Beyotime, Shanghai, China, Cat. No. S0101S). MDA content in serum and liver was measured using a kit (Nanjing Jiancheng, Nanjing, China, Cat. No. A003-1-2). TG content in feces and liver was measured using a kit (Nanjing Jiancheng, Nanjing, China, Cat. No. A110-1-1). TC content in feces and liver was measured using a kit (Nanjing Jiancheng, Nanjing, China, Cat. No. A111-1-1). BCA content in feces and liver was measured using a BCA Protein Assay Kit (Thermo Scientific, Waltham, MA, USA, Cat. No. 23225).

### 2.4. Total RNA Sequence Analysis

The experimental groups were defined as Normal Control (NC), Model Control (MC), and High-Dose LPPO (HD), with three biological replicates per group (*n* = 3). Total RNA was extracted from HepG2 cells using BS258A Total RNA extraction reagent (Biosharp), following the manufacturer’s instructions. The concentration and purity of total RNA were determined using a NanoDrop spectrophotometer (Thermo Scientific NanoDrop one, Thermo Fisher Scientific, Waltham, MA, USA), while RNA integrity was assessed through either RNA-specific agarose gel electrophoresis or the Agilent Fragment Analyzer 5400 system (Agilent Fragment Analyzer 5400, Agilent Technologies Inc., Santa Clara, CA, USA).

mRNA enrichment was performed using the NEBNext^®^ Poly(A) mRNA Magnetic Isolation Module (New England Biolabs Inc., Ipswich, MA, USA) with Oligo(dT) magnetic beads. The enriched mRNA was then randomly fragmented using divalent cations (ion interruption method) with the NEBNext Ultra II RNA Library Prep Kit for Illumina (New England Biolabs Inc., Ipswich, MA, USA). Using the fragmented mRNA as a template, double-stranded cDNA was synthesized with random oligonucleotides as primers (for strand-specific library construction, dUTP replaced dTTP in the dNTP mix during second-strand synthesis). The double-stranded cDNA was purified, followed by end repair, A-tailing, and adapter ligation. cDNA fragments of approximately 400–500 bp were size-selected using AMPure XP beads (Beckman Coulter, Beverly, CA, USA), PCR-amplified, and the PCR products were purified again with AMPure XP beads to obtain the final library. Library fragment sizes were analyzed using an electrophoresis-based instrument such as the LabChip GX. Total library concentration was measured using PicoGreen, and the effective library concentration was quantified by qPCR. Libraries were sequenced on the Illumina platform.

For the bioinformatic analysis, raw sequencing data were first filtered using fastp to remove adapter sequences and low-quality reads (average quality score < Q20), yielding high-quality clean data. The clean reads were subsequently mapped to the reference genome using HISAT2. Gene expression levels were quantified using HTSeq to obtain read counts, which were then normalized as FPKM (Fragments Per Kilobase of transcript per Million fragments mapped). Differential expression analysis between groups was performed using the DESeq2 package. The magnitude of differences between samples was indicated by the adjusted *p*-value and Fold-change (FC). Differentially expressed genes (DEGs) were identified using a threshold of |log_2_FC| > 1 and adjusted *p* < 0.05. Finally, Gene Ontology (GO) and Kyoto Encyclopedia of Genes and Genomes (KEGG) pathway enrichment analyses for the DEGs were conducted utilizing the clusterProfiler package, with statistical significance defined as a *p* < 0.05.

### 2.5. Molecular Docking Analysis

Target proteins (ALOX5, NOS2, and CD274) and ligands (quercetin) were retrieved from the PDB and the PubChem database (https://pubchem.ncbi.nlm.nih.gov/, accessed on 15 August 2026). SiteMap was utilized to predict potential docking sites between the protein and ligand. Based on the predictions, appropriate docking pockets were selected according to the reference scores. Docking simulations were performed using AutoDock Vina (version 1.2.3, The Scripps Research Institute, La Jolla, CA, USA) to calculate binding energies and visualize interaction sites [28].

### 2.6. Network Pharmacology Analysis

#### 2.6.1. Screening of Metabolite Targets Based on LC-MS Analysis

Using databases such as TCMSP (TCMSP, http://tcmspw.com/tcmspsearch.php, accessed on 18 August 2026), Swiss Target Prediction (http://www.swisstargetprediction.ch/, accessed on 18 August 2026), HERB (http://herb.ac.cn/, accessed on 19 August 2026), BATMAN-TCM (http://bionet.ncpsb.org.cn/batman-tcm/, accessed on 19 August 2026), the SMILES structures of compounds identified by LC-MS analysis were input to predict their potential target proteins. To ensure species consistency with the human-derived HepG2 cell model used for transcriptomics and molecular docking, all retrieved targets were uniformly mapped to Homo sapiens gene symbols using the UniProt database. Targets with a predicted probability >0.5 were screened, uniformly converted to the ‘symbol’ format, and used to derive the comprehensive list of active ingredient targets.

#### 2.6.2. Screening of MASLD-Related Targets 

To identify MASLD-related targets, this study utilized “MASLD” as a keyword to perform searches across multiple databases, including GeneCards (https://www.genecards.org, accessed on 20 August 2026), DrugBank (https://www.drugbank.ca/, accessed on 20 August 2026), Online Mendelian Inheritance in Man (OMIM, https://omim.org, accessed on 20 August 2026), and the Comparative Toxicogenomics Database (CTD, https://ctdbase.org, accessed on 20 August 2026). All retrieved disease targets were similarly filtered and mapped to Homo sapiens orthologs to align with the metabolite targets. This approach enabled the identification of target molecules associated with the pathogenesis of MASLD.

#### 2.6.3. Protein Interaction Network Assembly 

STRING database (https://string-db.org/, accessed on 21 August 2026) was utilized to obtain the intersection of targets identified from LPPO metabolites, MASLD, and differentially expressed genes in the HepG2 cell transcriptome, which were subsequently employed for protein–protein interaction (PPI) analysis. Then, Cytoscape3.7.2 received the results for network topology analysis, core target screening, and protein–protein interaction (PPI) network building. 

#### 2.6.4. Core Target Biofunctional Enrichment Analysis 

R software (version 4.1.0) was used to conduct Gene Ontology (GO) term, Kyoto Encyclopedia of Genes and Genomes pathway enrichment analyses (KEGG) and Gene Set Enrichment Analysis (GSEA) on LPPO’s MASLD related targets. This study aims to elucidate the functions of these target proteins and their roles in signal transduction, as well as to identify key signaling pathways.

### 2.7. Western Blot Analysis 

For HepG2 cells, after induction with 1 mM FFA, 10 μM quercetin was added for co-incubation for 24 h [29]. Quercetin was used instead of the crude LPPO extract for the Western Blot analysis because it was identified as the core bioactive metabolite via our multi-omics analysis, allowing us to specifically validate the direct molecular target interactions predicted by network pharmacology. With a commercial kit (Solarbio, Beijing, China) used for extracting HepG2 cell proteins, the proteins were quantified by BCA assay, separated through SDS-PAGE, transferred to a PVDF membrane, and blocked with skim milk. The membranes were then incubated overnight at 4 °C with specific primary antibodies obtained from HuaBio (Hangzhou, China): anti-ALOX5 (Cat. No. HA723968, 1:1000), anti-NOS2 (Cat. No. HA721583, 1:1000), anti-CD274 (Cat. No. HA723510, 1:1000), and anti-GAPDH (Cat. No. ET1601-4, 1:10,000). Following thorough washing, the membranes were incubated with an appropriate HRP-conjugated secondary antibody (Cat. No. HA1005, HuaBio, 1:10,000) for 1 h at room temperature. The protein bands were subsequently visualized on a gel imaging system. Band grayscale values were analyzed using ImageJ (version 1.53k, National Institute of Health, Bethesda, MD, USA), with GAPDH as the reference [30].

### 2.8. Statistical Analysis 

In in vivo animal experiments, three mice were used per group (*n* = 3); in in vitro cell experiments, transcriptomic sequencing, and Western blot analyses, three biological replicates were set up per experimental group (*n* = 3). Data are presented as the mean ± standard deviation (mean ± SD). Statistical analyses were performed using IBM SPSS Statistics (version 27.0, IBM Corp., Armonk, NY, USA). Comparisons between two groups were conducted using a Student’s *t*-test. For comparisons among three or more groups, one-way analysis of variance (ANOVA) followed by Tukey’s post hoc test was employed. Statistical significance was determined with a *p*-value threshold of less than 0.05.

## 3. Results and Discussion

Throughout our analysis, multiple endpoints were assessed using specific pairwise comparisons (e.g., MC vs. NC, HD vs. MC). Hepatic lipid accumulation and redox status were treated as primary outcomes, whereas transcriptomic and metabolomic alterations were considered exploratory outcomes. 

### 3.1. LPPO Ameliorates MASLD in C57BL/6J Mice (JAX Substrain)

To validate the efficacy of LPPO, we conducted animal experiments. The therapeutic efficacy of LPPO was rigorously validated in C57BL/6J mice (JAX substrain) through a multi-dimensional assessment of systemic metabolism, hepatic pathology, and lipid excretion pathways. The following data sets provide robust evidence for the ameliorative effects of LPPO on HFD-induced MASLD.

#### 3.1.1. Systemic Lipid Profiles and Antioxidant Capacity

Dietary intervention with LPPO for 8 weeks resulted in a significant restoration of serum metabolic homeostasis. Compared with the model group (MC), LPPO dose-dependently reduced serum levels of LDL-C, TC, and TG. These changes were accompanied by a modulation of HDL-C levels (Figure 1A–D). LPPO significantly enhanced serum SOD activity while suppressing the accumulation of MDA, a critical marker of lipid peroxidation (Figure 1E,F). A marked reduction in serum TBA levels was observed in LPPO-treated mice, indicating improved bile acid circulation (Figure 1G).

#### 3.1.2. Hepatic Lipid Deposition and Oxidative Protection

The direct impact of LPPO on the liver was quantified through biochemical analysis of hepatic tissues. LPPO treatment led to a substantial decrease in hepatic TC and TG concentrations (Figure 1H,I). Similar to the serum results, LPPO significantly elevated hepatic SOD activity and lowered hepatic MDA levels (Figure 1J,K). Hepatic BCA levels remained stable across groups, suggesting that LPPO specifically targets lipid metabolism without compromising total protein integrity (Figure 1L).

#### 3.1.3. Inhibition of Intestinal Lipid Absorption

A key highlight of the in vivo study was the analysis of fecal matter, which revealed a potential mechanism for the hypolipidemic effect of LPPO. Analysis of fecal parameters showed a significant increase in the excretion of TC and TG (Figure 1N,O). Conversely, fecal BCA levels were found to be decreased in the LPPO groups (Figure 1M). These results strongly suggest that LPPO may function by inhibiting intestinal absorption of dietary lipids or by promoting bile acid excretion, thereby reducing systemic lipid load.

While our data demonstrates increased fecal TC and TG excretion, the mechanisms connecting LPPO administration, intestinal lipid absorption, fecal lipid/bile acid excretion, and hepatic steatosis represent preliminary hypotheses that require validation in adequately powered clinical cohorts. Furthermore, the reduced circulating bile acids could alternatively be explained by impaired hepatic bile acid synthesis or alterations in enterohepatic recycling, rather than solely increased excretion. It also remains to be determined whether the fecal lipid excretion effect is selectively targeting cholesterol absorption or if it represents a broader impairment of intestinal lipid digestion, which could potentially interfere with the absorption of fat-soluble vitamins and essential fatty acids.

#### 3.1.4. Histopathological Restoration 

The biochemical improvements in lipid metabolism were visually and quantitatively confirmed by liver histopathology, providing definitive evidence of LPPO’s efficacy. As shown in the H&E stained sections (Figure 2B), liver tissues from the model control (MC) group exhibited severe architectural disruption and macrovesicular steatosis, characterized by widespread “ring-like” vacuoles and a “hole-filled” appearance. To quantitatively evaluate the severity of hepatic steatosis and liver injury, the standard NAFLD Activity Score (NAS) system was applied (Supplementary Table S1). The MC group displayed a high comprehensive NAS score of 7 ± 0.71, driven by prominent macrovesicular steatosis, localized lobular inflammatory infiltrates, and typical hepatocellular ballooning (swollen hepatocytes with pale, rarefied cytoplasm). 

In contrast, dietary intervention with LPPO dose-dependently ameliorated these pathological lesions. In the LD group, the size and density of the lipid vacuoles were substantially reduced, accompanied by alleviated hepatocellular ballooning (NAS = 3.00 ± 0.71, *p* < 0.05). Remarkably, the HD group displayed a clearly defined, nearly normal hepatic architecture with a regular “plate-like” arrangement of hepatocytes. Neutral lipid vacuoles and inflammatory foci were virtually eliminated, dramatically reducing the comprehensive NAS score to baseline levels (0.00 ± 0.00, *p* < 0.05). Consistent with the H&E and NAS evaluations, Oil Red O staining (Figure 2A) demonstrated that the extensive, coalesced red lipid droplets in the model group were reduced to only scattered, sporadic red dots following LPPO intervention, confirming a robust clearance of neutral lipid accumulation. These systematic histopathological evaluations firmly corroborate that LPPO not only reduces systemic lipids but directly protects against high-fat-induced hepatocyte structural damage and steatohepatitis. 

To determine whether the decrease in steatosis might be secondary to a reduction in effective energy intake or potential toxicity rather than a specific pharmacological effect, food intake, water intake, and body weight were continuously monitored. As shown in Supplementary Figure S3A,B, there were no significant differences in daily food and water intake among the MC, LD, and HD groups, indicating that LPPO treatment did not affect the animals’ appetite or drinking behavior. However, the body weight data (Supplementary Figure S3C) revealed that high-dose LPPO intervention attenuated the excessive weight gain induced by the high-fat diet compared to the MC group. These results confirm that the amelioration of hepatic steatosis is a specific metabolically driven pharmacological effect rather than a mere consequence of reduced caloric intake.

### 3.2. LPPO Attenuates Intracellular Lipid Accumulation and Oxidative Stress

To determine whether LPPO derived from lychee affects lipid metabolism in HepG2 cells, we conducted relevant experiments using LPPO obtained from FZX. The cytotoxicity screening (Figure 3A) revealed that HepG2 cell viability decreased in a concentration-dependent manner as the test concentration increased from 0 to 250 ng/mL, with viability remaining relatively high only at 50 ng/mL and 100 ng/mL. On this basis, 50 ng/mL (LD group) and 100 ng/mL (HD group) were selected as the safe working concentrations for all subsequent assays. In the steatotic model (MC group), morphological observation (Figure 3B, 20×) revealed pronounced accumulation of densely packed red lipid droplets within the cytoplasm, while intracellular TC (Figure 3C) was significantly elevated by 76.02% and intracellular TG (Figure 3D) were significantly elevated by 103.05% compared with the NC group, confirming the successful establishment of the lipid-overload phenotype.

Both LD and HD treatments markedly suppressed lipid droplet deposition and dose-dependently restored intracellular TC and TG levels, with the HD group exhibiting a greater magnitude of reduction than the LD group and TG levels approaching the NC baseline, indicating a robust lipid-lowering activity of LPPO. This hypolipidemic effect is consistent with that reported for procyanidin A2. He et al. demonstrated that this polyphenolic constituent derived from litchi pericarp phosphorylates AMPK and down-regulates HMG-CoA reductase, FAS, and SREBP-1c, thereby significantly suppressing oleic acid-induced lipid accumulation in HepG2 cells [31]. The present results are also similar to those reported for grape peel polyphenols [32], Coffea arabica pulp polyphenols (containing chlorogenic acid, catechin and epicatechin) [33], and Idesia polycarpa fruit polyphenols [34], which respectively reduced TG and ROS levels in sodium palmitate-treated HepG2 cells via the AMPK/mTOR/AKT pathway, lowered intracellular TC and TG contents through up-regulation of PPARα and down-regulation of PPARγ, and ameliorated lipid metabolic dysfunction in oleic acid-induced HepG2 cells through the PPARα pathway.

With regard to oxidative stress (Figure 3E,F), SOD activity in the MC group was markedly decreased compared with the NC group, and the SOD inhibition rate—reflecting the capacity of SOD to scavenge superoxide anions—exhibited a concordant downward trend, collectively indicating impairment of the endogenous antioxidant enzyme system; concomitantly, the lipid peroxidation product MDA was significantly elevated in the MC group, demonstrating severe disruption of the cellular antioxidant defense system during model induction. Following LD and HD treatment, both SOD activity and SOD inhibition rate were restored in a dose-dependent manner, whereas MDA levels were dose-dependently suppressed, with the HD group showing the most pronounced recovery and MDA levels approaching the NC baseline. This antioxidant-rescue pattern is highly consistent with that reported for blueberry leaf polyphenols. Blueberry leaves can activate the AMPK/PGC-1α/SIRT3 signaling pathway and enhance mitochondrial antioxidant capacity, thereby alleviating hepatic steatosis and oxidative stress in both HepG2 cells and MASLD model mice [22]. It is also similar to the findings reported for berry and grape pomace polyphenols [35], demonstrating that polyphenol-rich fluid extracts from haskap berries, bitter cherries and red grape pomace significantly reduced MDA, ROS and NO levels and inhibited lipid droplet formation in free fatty acid-stressed HepG2 cells.

Taken together, the HD group consistently outperformed the LD group across all indices, including TC, TG, SOD activity, SOD inhibition rate, and MDA, restoring lipid metabolism and redox homeostasis toward the NC-level baseline. These findings indicate that LPPO exerts a marked, dose-dependent dual hepatoprotective effect on lipid metabolism and oxidative stress, in good agreement with the “lipid-regulating–antioxidant” synergistic paradigm, consistently observed for dietary polyphenols in recent years in HepG2-based MASLD models.

### 3.3. Transcriptomic Analysis of Pathways and Gene Expression Regulated by LPPO

To systematically elucidate the molecular mechanisms underlying LPPO’s role in alleviating hepatic steatosis, transcriptomic sequencing was performed on NC, MC, and HD groups in cell experiments. Global gene expression analysis via volcano plots (Figure S2A–C) and KEGG enrichment (Figure S2D,E) demonstrated a significant shift in the transcriptomic landscape following FFA induction and LPPO intervention, underscoring its systemic regulatory capacity.

Hierarchical clustering heatmap combined with group boxplots (Figure 4A) revealed high intra-group consistency and significant inter-group divergence. The high degree of reproducibility within groups and the distinct separation between groups validated the robustness of the dataset for downstream analysis. KEGG enrichment analysis identified Arachidonic acid metabolism as the core regulatory pathway (Figure 4B). This was primarily manifested in the modulation of unsaturated fatty acid metabolic pathways, providing a singular and prominent mechanism for the observed hypolipidemic effects.

The interactive heatmap (Figure 4C) illustrated the consistent upward or downward trends of DEGs across the experimental groups. Quantification of Differentially Expressed Genes (DEGs): A lollipop plot (Figure 4D) visualized the specific Fold Change (|log_2FC| > 1, *p* < 0.05) of key DEGs. The analysis revealed a profound upregulation of GMPR (log_2_FC = 4.78), alongside notable increases in key regulators of lipid metabolism and cellular stress, such as ENC1 (log_2_FC = 1.26) and PLA2G3 (log_2_FC = 1.14). Conversely, several metabolic and signaling-related markers [36,37], most distinctly BCKDHA (log_2_FC = −4.79) and PDE6G (log_2_FC = −2.74), were significantly suppressed. Specifically, the “dual-regulation” of redox homeostasis was evidenced by the upregulation of ENC1 (log_2_FC = 1.26) and MPV17L2 (log_2_FC = 1.01), alongside the downregulation of DUOX1 (log_2_FC = −1.06) [38,39].

These trends collectively demonstrate that LPPO exerts effects on AMPK and energy metabolism, lipid synthesis and metabolism, antioxidants and stress response, endoplasmic reticulum stress and autophagy, suggesting its potential to promote metabolic reprogramming by enhancing lipid utilization and mitigating oxidative damage.

### 3.4. Network Pharmacology Data Analysis

Based on UPLC-MS/MS analysis, Table 1 summarizes the polycyclic phenolic compounds identified in litchi pericarp (Feizixiao cultivar). The major components included flavan-3-ols such as (-)-epicatechin, catechin, epigallocatechin, and afzelechin; oligomeric proanthocyanidins like procyanidins A2, B1, B3, and C1; flavonol glycosides including rutin, kaempferol-3-O-rutinoside, and isorhamnetin-3-rutinoside; and flavonoid aglycones such as quercetin, fisetin, and isorhamnetin. Other phenolic compounds comprised phloretin, phlorhizin, caftaric acid, and isoferulic acid. These observations are consistent with the experimental data reported by other researchers [10,11,12].

According to Table 2, quercetin has the highest number of targets at 431, surpassing catechin, isorhamnetin, rutin, and (-)-epicatechin, which have 215, 172, 122, and 118 targets, respectively. Therefore, quercetin was prioritized as the core metabolite for subsequent molecular docking and Western blot validation experiments. Quantitative analysis revealed that quercetin is present at an abundance of 1.2 mg/g in the LPPO, further justifying its selection as the key bioactive representative for subsequent in vitro mechanistic validation.

To identify potential targets associated with the FFA versus LPPO comparison, we first intersected disease-related targets, metabolite-related targets, and differentially expressed genes. As shown in Figure 5A, four common targets were identified: ALOX5, NOS2, SLC5A11, and CD274 (PD-L1). To further prioritize the common targets, we imported them into STRING for protein–protein interaction (PPI) analysis (Figure 5B). The PPI network showed that ALOX5, NOS2, and CD274 were connected, whereas SLC5A11 was not connected with the other targets (Figure 5B). Among the interacting pairs, CD274-NOS2 had the highest combined score (0.398), followed by ALOX5-NOS2 (0.284) and ALOX5-CD274 (0.207). Therefore, ALOX5, NOS2, and CD274 were prioritized as core targets for subsequent molecular docking and Western blotting validation.

GO enrichment analysis was then performed to characterize the biological functions represented by the core targets. The analysis identified 145 overlapping terms (Figure 5C). The leading terms included regulation of cytokine production involved in inflammatory response, regulation of insulin secretion, leukotriene A4 biosynthetic process, lipoxin biosynthetic process, positive regulation of leukocyte-mediated cytotoxicity, and negative regulation of tumor necrosis factor superfamily cytokine production. The enrichment of leukotriene A4 and lipoxin biosynthesis was consistent with the role of ALOX5 in arachidonic acid-derived lipid mediator metabolism [40,41]. The nitric oxide synthase-related and arginine-associated signals were consistent with the role of NOS2/iNOS in arginine metabolism, nitric oxide production, and inflammatory macrophage activation [42,43]. Because amino acid transport and metabolism are closely related to myeloid immune function, these arginine-related results also supported an immunometabolic interpretation [42,44]. In addition, the CD274-related terms supported the possible involvement of PD-L1-mediated immune regulation [45].

KEGG pathway analysis identified 24 pathways related to the core targets (Figure 5D). Toxoplasmosis met the FDR < 0.05 criterion (FDR = 0.014), with ALOX5 and NOS2 as overlapping genes. Other nominally enriched pathways included arginine biosynthesis, arginine and proline metabolism, ovarian steroidogenesis, arachidonic acid metabolism, Fc epsilon RI signaling pathway, Leishmaniasis, Pertussis, and PD-L1 expression and PD-1 checkpoint pathway in cancer. Although only Toxoplasmosis remained significant after FDR correction, the combined GO and KEGG results were consistent with involvement of inflammatory lipid mediator metabolism [40,41], arginine/NO metabolism [42,43], and PD-L1-related immune regulation [45].

GSEA was performed to evaluate pathway-level changes across the full ranked gene list from the FFA versus LPPO comparison. As shown in Figure 6A, 21 KEGG pathways reached nominal significance (*p* < 0.05), whereas no pathway passed the FDR < 0.25 threshold. The most negative NES values were observed for retrograde endocannabinoid signaling (NES = −1.508), dopaminergic synapse (NES = −1.483), tyrosine metabolism (NES = −1.492), and relaxin signaling pathway (NES = −1.371), indicating enrichment toward the FFA-associated end of the ranked list. In contrast, Toll-like receptor signaling pathway showed a positive NES (NES = 1.438, *p* = 0.016, leading-edge count = 14), indicating enrichment toward the LPPO-associated end. 

The classical GSEA curves further showed the distribution of representative gene sets. The three GO gene sets shown in Figure 6B, including odorant binding, structural constituent of tooth enamel, and negative regulation of ATPase-coupled calcium transmembrane transport, displayed positive running enrichment scores concentrated near the beginning of the ranked list. The three KEGG gene sets shown in Figure 6C, including retrograde endocannabinoid signaling, dopaminergic synapse, and tyrosine metabolism, displayed negative running enrichment scores toward the end of the ranked list. The positive enrichment of Toll-like receptor signaling was consistent with evidence that TLR4 activation can reprogram macrophage fatty acid and sphingolipid metabolism during inflammatory activation [46] and with the role of NF-κB in inflammatory and immune signaling [47]. However, because none of the KEGG-GSEA pathways passed FDR correction, these observations were considered exploratory.

Overall, the network pharmacology analysis prioritized ALOX5, NOS2, and CD274 as core targets associated with the FFA versus LPPO comparison, whereas quercetin was prioritized as the core metabolite. GSEA provided exploratory, direction-specific context at the transcriptome level. This interpretation is consistent with recent work emphasizing that network pharmacology is strengthened by integration with transcriptomics, metabolomics, molecular docking, and experimental validation [48,49,50]. Accordingly, quercetin, ALOX5, NOS2, and CD274 were selected for further validation to determine whether inflammatory lipid mediator metabolism, arginine/NO metabolism, and PD-L1-related immune regulation contribute to the biological differences between FFA and LPPO.

### 3.5. Molecular Docking and Target Validation

Based on network pharmacology results, this study identified ALOX5, NOS2, and CD274 as potential target molecules for LPPO activity, with quercetin serving as a key metabolite. To validate the effects of quercetin on these three targets, we conducted molecular docking studies.

Quercetin is positioned within a defined pocket of ALOX5 and is surrounded by GLN549, VAL243, THR366, ARG370, ARG457, ALA453, and PHE450. The enlarged view shows several short-range contacts of approximately 2.4–3.9 Å. The polar side chains of GLN, THR, and ARG are compatible with hydrogen-bonding or electrostatic contributions, whereas PHE450 and the aromatic rings of quercetin may contribute π-related and hydrophobic stabilization (Figure 7A).

Quercetin occupies a pocket formed by SER242, LEU209, TRP194, ALA197, ARG199, TYR489, and PHE369. The interaction distances shown are mainly within approximately 3.1–4.0 Å. The aromatic residues PHE369, TYR489, and TRP194 can support π-π or π-alkyl contacts with the flavonoid scaffold, while SER242 and ARG199 may provide polar stabilization. The extensive distribution of contacts around both aromatic rings and hydroxyl-bearing regions of quercetin is consistent with a well-packed docking pose (Figure 7B).

Quercetin is embedded in a CD274 pocket containing TYR56, ASN63, GLN66, MET115, ILE116, ALA121, ASP122, and TYR123. Multiple contacts are displayed at approximately 3.0–4.1 Å. Polar residues ASN63, GLN66, and ASP122 may interact with the hydroxyl and carbonyl groups of quercetin, whereas TYR56 and TYR123 may provide aromatic stabilization. The pose indicates complementary polar and hydrophobic contacts across the ligand-binding region (Figure 7C).

Figure 7D displays the 3,3′,4′,5,7-pentahydroxyflavone scaffold of quercetin. Its multiple hydroxyl groups and conjugated aromatic system explain its capacity to participate in hydrogen bonding, electrostatic interactions, π-stacking, and hydrophobic contacts within protein pockets. Figure 7E shows the result of Western blot. Western blot analysis revealed that the basal protein expression levels of ALOX5 (78 kDa) and NOS2 (130 kDa) were extremely low in the Control HepG2 cells, presenting as faint bands. Following the modeling induction, the protein expressions of ALOX5 and NOS2 were significantly upregulated in the Model group. However, treatment with Quercetin resulted in a marked decrease in the protein levels of both ALOX5 and NOS2. These results indicate that Quercetin can effectively suppress the overexpression of inflammation-related proteins in HepG2 cells. Regarding CD274/PD-L1, due to its extensive intracellular glycosylation, it typically resolves as a 45–55 kDa smeared band (smear) in immunoblots. Our experimental results demonstrated a moderate basal expression of PD-L1 in the control cells. Whereas in the model group, lipid accumulation might have activated the STAT3 and other related signaling pathways, leading to a robust upregulation of PD-L1 expression (characterized by the most intense and thickest bands). Notably, quercetin administration effectively reversed this aberrant upregulation of PD-L1.

The data of Table 3 show favorable predicted binding for all three complexes, with binding energies below −9.0 kcal/mol. The most favorable score was obtained for NOS2, indicating that NOS2 is the leading predicted quercetin-binding target among the three. CD274 and ALOX5 also displayed stable pocket occupancy and multiple short-range contacts. Collectively, these results support a multi-target model in which quercetin may interact with inflammatory lipid metabolism, oxidative/nitrosative stress, and immune-checkpoint regulation. 

Quercetin could have an impact on NOS2/NO-ROS/NF-κB-HIF-1α/CD274 axis. NOS2 encodes inducible nitric oxide synthase, which generates large amounts of nitric oxide under inflammatory conditions. Sustained NO production, together with ROS, contributes to nitrosative and oxidative stress and can intersect with NF-κB and HIF signaling. NF-κB, HIF-1α, JAK/STAT, and other stress-responsive transcriptional programs are recognized regulators of CD274/PD-L1 expression [51,52]. Therefore, the LPPO experimentally reduced the abundance or activity of NOS2; a plausible mechanistic explanation for this is that the NOS2/NO–ROS inflammatory network was inhibited, subsequently leading to a reduced activation level of the PD-L1-promoted transcriptional pathway.

Quercetin has the potential to influence ALOX5/5-HETE-LTB4 immunometabolic pathway. ALOX5 is the key enzyme of the 5-lipoxygenase branch of arachidonic-acid metabolism. It generates 5-HETE and leukotrienes, including LTB4, which function as pro-inflammatory lipid mediators. Recent experimental work demonstrated that the ALOX5/5-HETE axis can increase PD-L1-positive immunosuppressive M2-like glioma-associated macrophages and promote PD-L1 expression through Nrf2-related signaling [53]. A separate 2023 study showed that tumor-cell-derived ALOX5/LTB4 recruits M2 macrophages through BLT1/BLT2 and PI3K signaling [54]. These findings support an ALOX5-driven lipid-metabolism/immune-microenvironment pathway. Accordingly, LPPO-associated ALOX5 downregulation could reduce 5-HETE/LTB4 signaling, weaken M2-type immunosuppressive remodeling, and indirectly reduce PD-L1-associated immune suppression.

### 3.6. Mechanism Analysis

Figure 8 provides a detailed illustration of the molecular mechanism model by which LPPO alleviates hepatic steatosis. Rather than a definitive pathway, this model represents an integrated hypothesis derived from our transcriptomic profiling, in vitro and in vivo biochemical assays, and in silico molecular docking predictions. Our in vivo data confirm that LPPO robustly reverses lipid-induced damage and oxidative stress, which is consistent with previous studies on polyphenols [55,56,57]. In the models proposed below, we explicitly separate these direct experimental observations from computational predictions and literature-inferred mechanisms.

#### 3.6.1. Suppression of Metabolic Inflammation via the Arachidonic Acid Pathway

Experimentally, our transcriptomic KEGG enrichment identified “Arachidonic acid metabolism” as a core regulatory pathway. Specifically, transcriptomic data showed a significant downregulation of ALOX12B and an upregulation of PLA2G3, while Western blot analysis confirmed that quercetin treatment resulted in a marked decrease in ALOX5 protein levels under lipotoxic conditions. Complementary to these experimental observations, our molecular docking models predict that quercetin exhibits a strong binding affinity for the ALOX5 active pocket (−9.102 kcal/mol). 

Based on these findings and existing literature, we hypothesize a downstream mechanism. Chronic FFA exposure is known to trigger basal inflammation driven by lipid mediators [58,59,60]. Because we observed a dual blockade of ALOX5 and ALOX12B, we assume this suppression limits the generation of pro-inflammatory lipid peroxides and leukotrienes (e.g., 5-HETE and LTB4) at the source. While we did not directly measure these lipid mediators or macrophage polarization in this study, the observed ALOX5 downregulation suggests a theoretical mechanism whereby LPPO weakens downstream M2-type immunosuppressive remodeling, a premise consistent with established roles of the arachidonic acid cascade [61,62]. 

#### 3.6.2. Dual-Regulation of Redox Homeostasis and Nitrosative Stress

Our experimental data demonstrate a profound phenotypic restoration of SOD activity and suppression of MDA levels in both in vitro and in vivo models. At the molecular level, Western blot analysis confirmed that quercetin effectively suppresses the aberrant overexpression of NOS2. Concurrently, transcriptomic profiling showed that LPPO significantly suppresses DUOX1 (log_2_FC = −1.06) while upregulating ENC1 (log_2_FC = 1.26) and MPV17L2 (log_2_FC = 1.01). This upregulation preserves mitochondrial membrane potential and facilitates the conversion of harmful oxidative metabolites (e.g., S_2_O_3_^2−^) to H_2_O, effectively shielding organelles from oxidative stress. Computationally, our molecular docking models predict a strong binding affinity between quercetin and NOS2 (binding energy = −9.46 kcal/mol). 

Integrating these data with existing literature, we propose a “dual-regulation” hypothesis. The combined inhibition of the NOS2/NO and DUOX1/ROS axes efficiently shields organelles from oxidative and nitrosative stress. The imbalance between ROS production, NO generation, and antioxidant scavenging is a recognized primary driver of MASLD progression [63,64]. Since DUOX1 is a major source of endogenous ROS [65,66], its transcriptomic suppression, combined with the protein-level suppression of NOS2, forms the basis of our assumed mechanism. While molecular docking suggests potential direct NOS2 engagement by quercetin, the suppression may also be transcriptionally regulated; regardless, we hypothesize that this combined inhibition efficiently shields organelles from oxidative and nitrosative stress. 

#### 3.6.3. Immune-Metabolic Regulation via the CD274 (PD-L1) Axis

Experimentally, we observed a robust upregulation of PD-L1 (CD274) protein in the model group, which was effectively reversed following quercetin administration as validated by Western blot. In silico, molecular docking demonstrated that quercetin could stably bind to the CD274 pocket (−9.32 kcal/mol) through multiple hydrogen bonds and aromatic stabilizations. 

Mechanistically, this observed downregulation could occur through two assumed pathways. First, the literature establishes that CD274 expression is highly responsive to inflammatory and stress-related transcriptional programs, including NF-κB and HIF-1α, which are driven by NO and ROS [67,68]. Therefore, the PD-L1 reduction may be a secondary consequence of the upstream NOS2/NO-ROS axis suppression we observed. Alternatively, our docking results suggest a second hypothesis: quercetin might directly physically interact with CD274. While our current experimental design cannot isolate which mechanism dominates, both assumed pathways converge on the reduction in PD-L1-associated immune suppression in the hepatic microenvironment, aligning with evidence that quercetin can cooperate with immune-checkpoint regulation [69]. 

#### 3.6.4. Restoration of Energy Homeostasis and Lipid Remodeling

Experimentally, our transcriptomic profiling revealed a shift in metabolic gene expression under conditions of chronic lipotoxicity. We observed a massive down regulation of BCKDHA and PPP1R3E, alongside a profound up regulation of GMPR, IDI1, and MSMO1 in the LPPO-treated group. Concurrently, our in vivo biochemical assays demonstrated that LPPO significantly promotes the fecal excretion of TC, leading to a marked decline in net hepatic and systemic cholesterol levels. 

Integrating these transcriptomic and physiological data with existing literature, we hypothesize that LPPO induces a profound “metabolic resource reallocation”. The observed changes in BCKDHA, PPP1R3E, and GMPR suggest a diversion of cellular energy from high-stress catabolic pathways toward active nucleotide metabolism and survival mechanisms. Furthermore, based on the established roles of IDI1 and MSMO1 in the mevalonate pathway, we assume LPPO actively promotes benign intracellular lipid remodeling via localized de novo cholesterol biosynthesis. Consistent with established literature on cholesterol homeostasis [70], this targeted metabolic shift likely provides essential lipid building blocks for cellular membrane repair to counteract lipid-induced damage. Ultimately, we propose that LPPO balances this localized compensatory lipid synthesis (for membrane repair) with robust systemic lipid clearance, efficiently restoring overall energy and lipid homeostasis. 

#### 3.6.5. Systemic Clearance and Intestinal Modulation

The profound histological repair and reduction in hepatic steatosis observed in C57BL/6J mice (JAX substrain) indicate that the therapeutic efficacy of LPPO extends beyond localized hepatic regulation to encompass systemic lipid clearance. The significant elevation in fecal TG and TC excretion in LPPO-treated cohorts strongly suggests that LPPO exerts modulatory effects within the gastrointestinal tract. This aligns with findings that quercetin is actively associated with improvements in gut microbiota and bile-acid metabolism [71,72,73]. By potentially inhibiting intestinal lipase activity or interfering with bile acid-mediated lipid emulsification, LPPO restricts dietary lipid absorption. It is a systemic clearance mechanism that is intimately linked to the modulation of intestinal microecology and plasma cholesterol homeostasis [74]. Ultimately, this multi-organ synergistic paradigm—mitigating hepatic lipid accumulation via the ALOX5/NOS2/CD274 regulatory network and transcriptome-level metabolic reprogramming, while accelerating systemic lipid clearance through enhanced fecal excretion—constitutes the comprehensive mechanism by which LPPO alleviates MASLD.

While naturally occurring molecules are desirable, it is important to emphasize that treating MASLD requires a multimodal approach encompassing lifestyle modifications and pharmacological treatment. Furthermore, naturally occurring substances are not entirely free of side effects. Extensive preclinical and some clinical evidence suggest possible pharmacokinetic interactions for quercetin [75,76,77,78,79]. Currently, there are insufficient studies to support the safe use of oligomeric polyphenols derived from lychee pericarp, warranting further thorough investigation.

## 4. Conclusions

This study systematically demonstrates the potent hypolipidemic and hepatoprotective effects of LPPO derived from the Feizixiao cultivar against MASLD. In high-fat diet-fed mice and free fatty acid-induced HepG2 cells, LPPO significantly reduces hepatic steatosis, restores redox homeostasis, and accelerates systemic lipid clearance through enhanced fecal excretion. Integrated multi-omics, molecular docking, and Western blot analyses reveal a multi-target metabolic reprogramming paradigm driven by key metabolites such as quercetin. Mechanistically, LPPO suppresses arachidonic acid-mediated inflammation by targeting ALOX5 and ALOX12B, and reduces oxidative and nitrosative stress via NOS2 and DUOX1 suppression, along with MPV17L2 upregulation. Notably, LPPO downregulates the CD274 (PD-L1) immune-checkpoint axis to reverse hepatic immunosuppression, while optimizing energy allocation and lipid remodeling through modulation of GMPR, BCKDHA, and IDI1. Collectively, these findings provide a robust theoretical and molecular basis for the valorization of litchi pericarp waste as a functional food ingredient for liver health management. 

Although these mechanistic insights are promising, several areas require further investigation. First, our in vitro cell models lacked an appropriate positive pharmacological control (such as standard statins or fibrates), which limits the ability to benchmark the absolute lipid-lowering efficacy of LPPO against established clinical agents. Future in vitro and in vivo studies should include positive control groups to comprehensively evaluate the comparative pharmacological potency of litchi pericarp polyphenols. Secondly, the in vivo MASLD phenotype in this study was driven by an accelerated 60% high-fat diet rather than a clinically reflective Western diet (which typically incorporates high fructose and cholesterol). Therefore, future in vivo investigations utilizing more physiologically relevant dietary models and prolonged intervention periods are warranted to fully translate these findings to human metabolic disease contexts. Thirdly, the marked increase in fecal triglyceride and cholesterol excretion following LPPO intervention suggests potential modulatory effects within the gastrointestinal tract. Future research should prioritize metagenomic analyses to examine the interactions between LPPO and the gut microbiome, with particular attention to how microbiota modulation contributes to systemic lipid clearance. Additionally, comprehensive pharmacokinetic profiling is needed to clarify the in vivo absorption, distribution, and metabolic fate of LPPO’s principal bioactive metabolites, especially quercetin and key procyanidins. Ultimately, rigorously designed clinical trials are necessary to assess the safety, bioavailability, optimal dosing strategies, and long-term therapeutic efficacy of LPPO in individuals with MASLD.

## Figures and Tables

**Figure 1:**
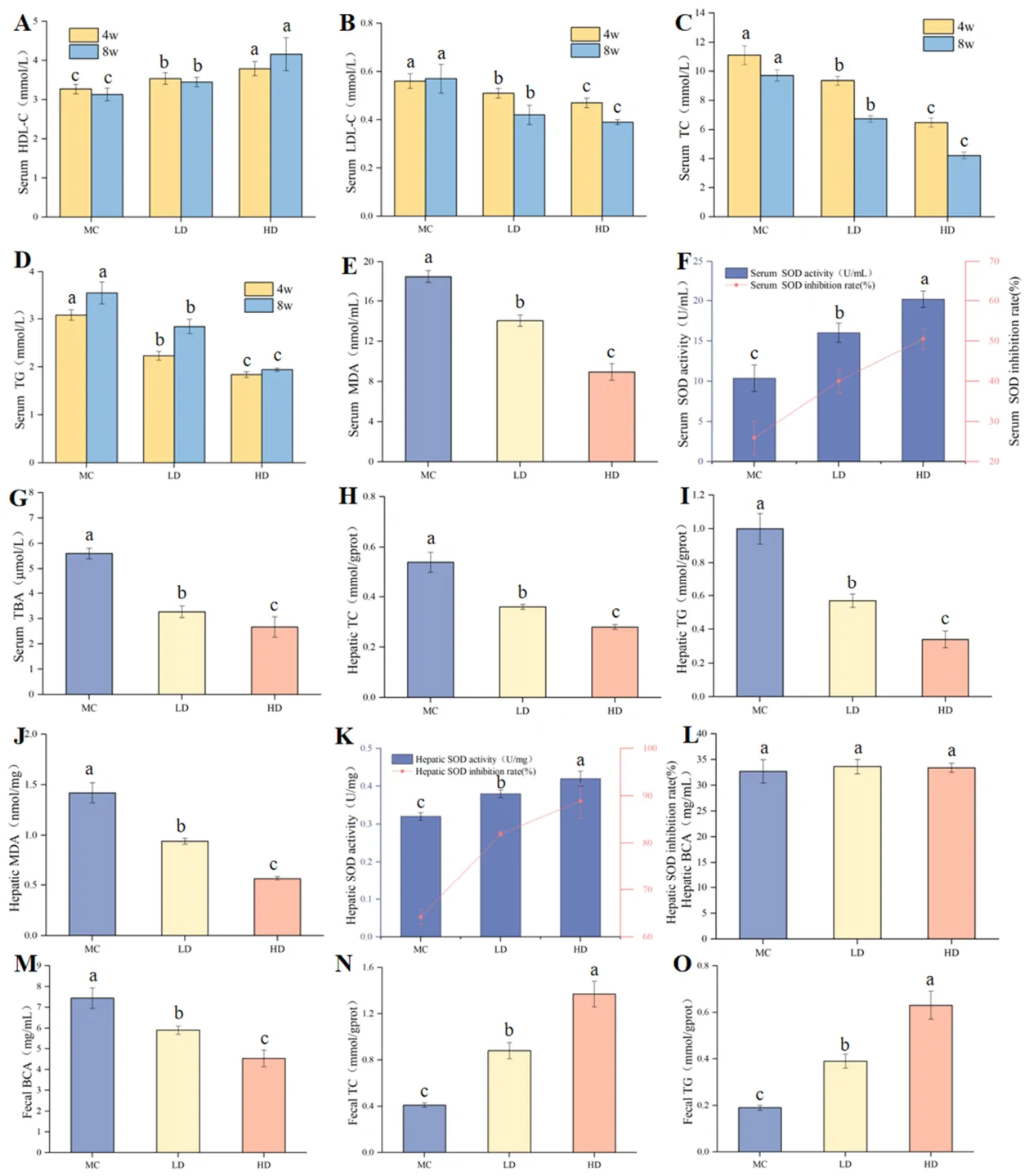
Systemic and hepatic effects of LPPO in C57BL/6J mice (JAX substrain). Serum HDL-C (**A**), LDL-C (**B**), TC (**C**), and TG (**D**) at 4 and 8 weeks. Serum MDA (**E**), SOD activity (**F**), and TBA (**G**). Hepatic TC (**H**), TG (**I**), MDA (**J**), SOD activity (**K**), and BCA protein (**L**). Fecal BCA (**M**), TC (**N**), and TG (**O**) levels. Different lowercase letters (a–c) above the bars indicate statistically significant differences among groups (*p* < 0.05).

**Figure 2:**
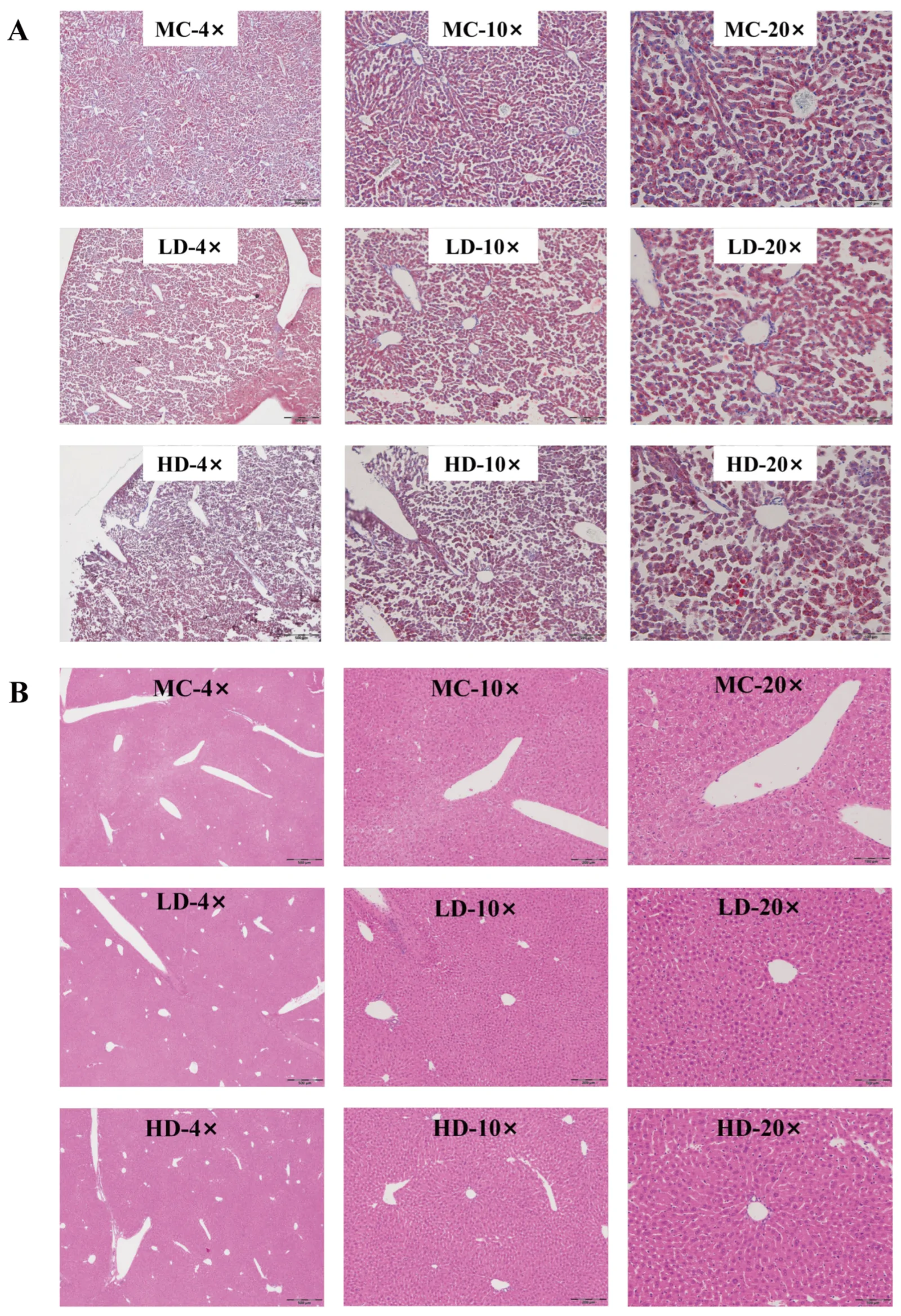
Histopathological assessment of liver tissue. (**A**) Oil Red O staining confirming reduction in neutral lipid droplets. (**B**) H&E staining showing vacuole reduction in LPPO-treated groups.

**Figure 3:**
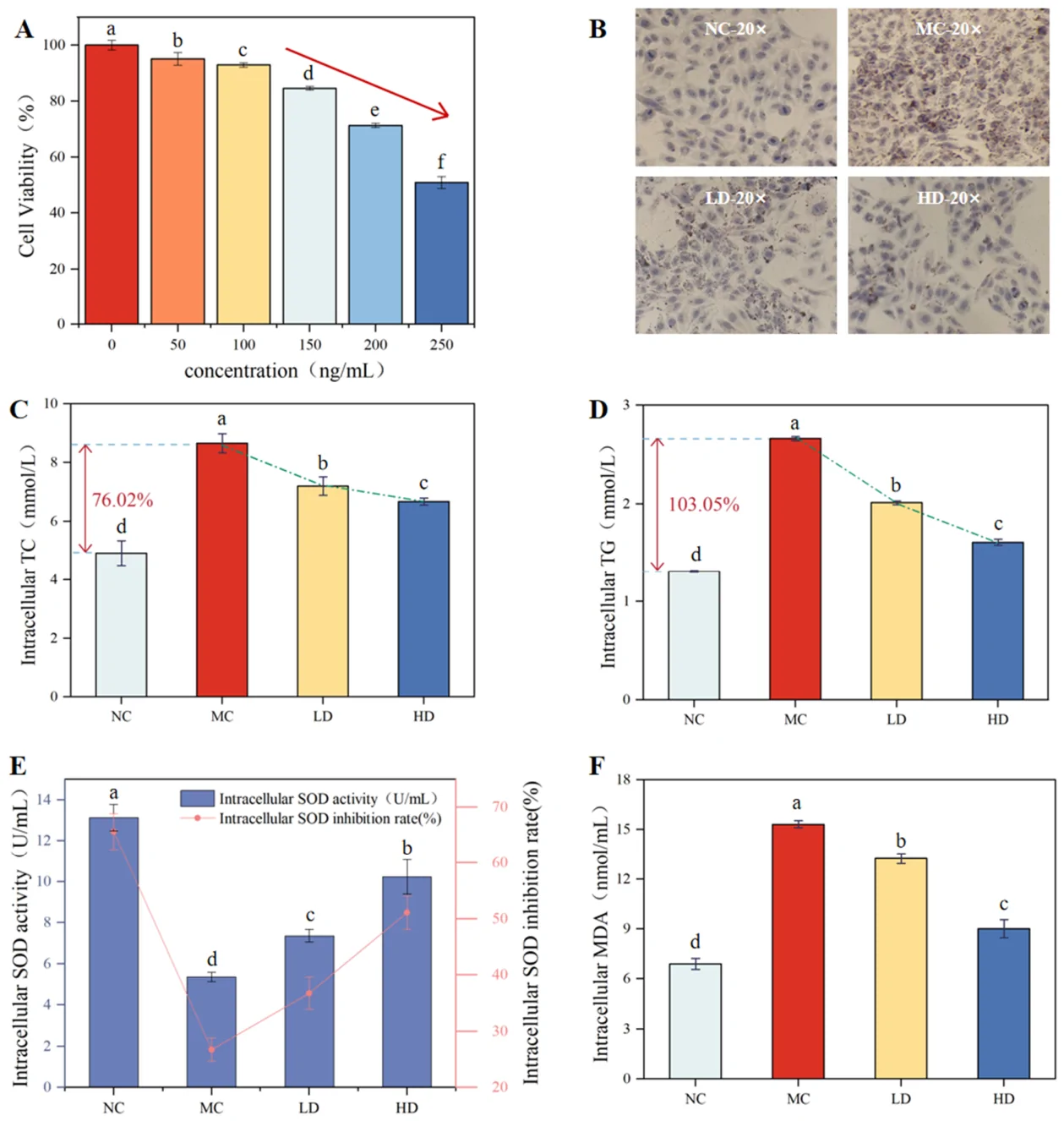
LPPO attenuates lipid accumulation and oxidative stress in HepG2 cells. (**A**) Cell viability determined by CCK-8 assay. (**B**) Oil Red O staining of HepG2 cells. Quantified intracellular TC (**C**) and TG (**D**) content. Intracellular SOD activity (**E**) and MDA levels (**F**). Data are presented as mean ± SD (*n* = 3). Different lowercase letters (a–f) above the bars indicate statistically significant differences among groups (*p* < 0.05). The red arrow in (**A**) indicates the concentration-dependent decline in cell viability, and the red double-headed arrows in (**C**,**D**) represent the percentage increase in the MC group compared with the NC group.

**Figure 4:**
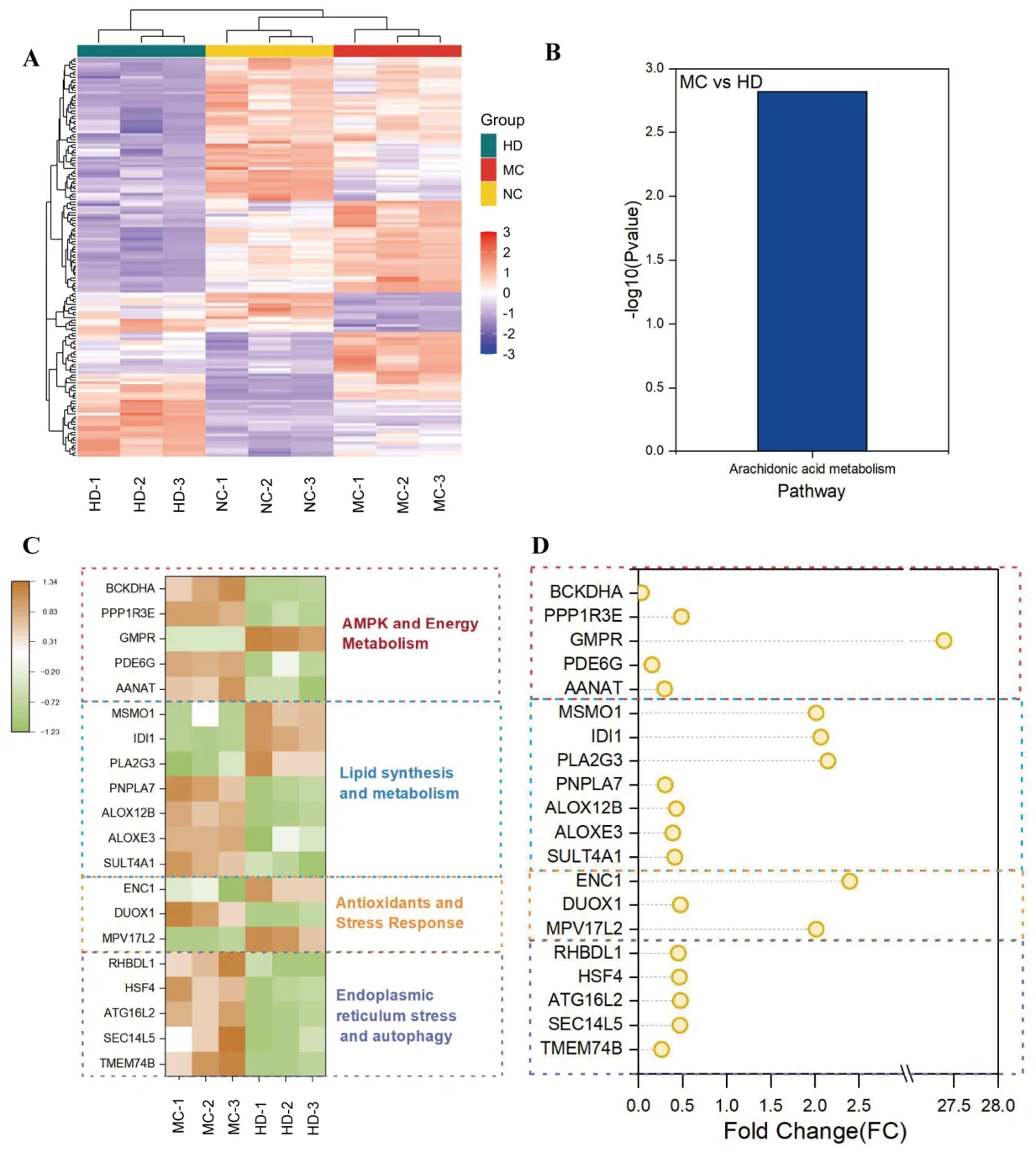
Transcriptomic analysis of pathways and gene expression regulated by LPPO. (**A**) Hierarchical clustering heatmap and gene clusters across NC, MC, and HD groups. (**B**) KEGG pathway enrichment identifying Arachidonic acid metabolism. (**C**) Functional heatmap. (**D**) Lollipop plot showing Fold Change (log_2_FC) of key DEGs. The colored dashed boxes group the genes into distinct functional categories, and the grey dashed lines in (**D**) serve as guide lines indicating the exact Fold Change value for each gene.

**Figure 5:**
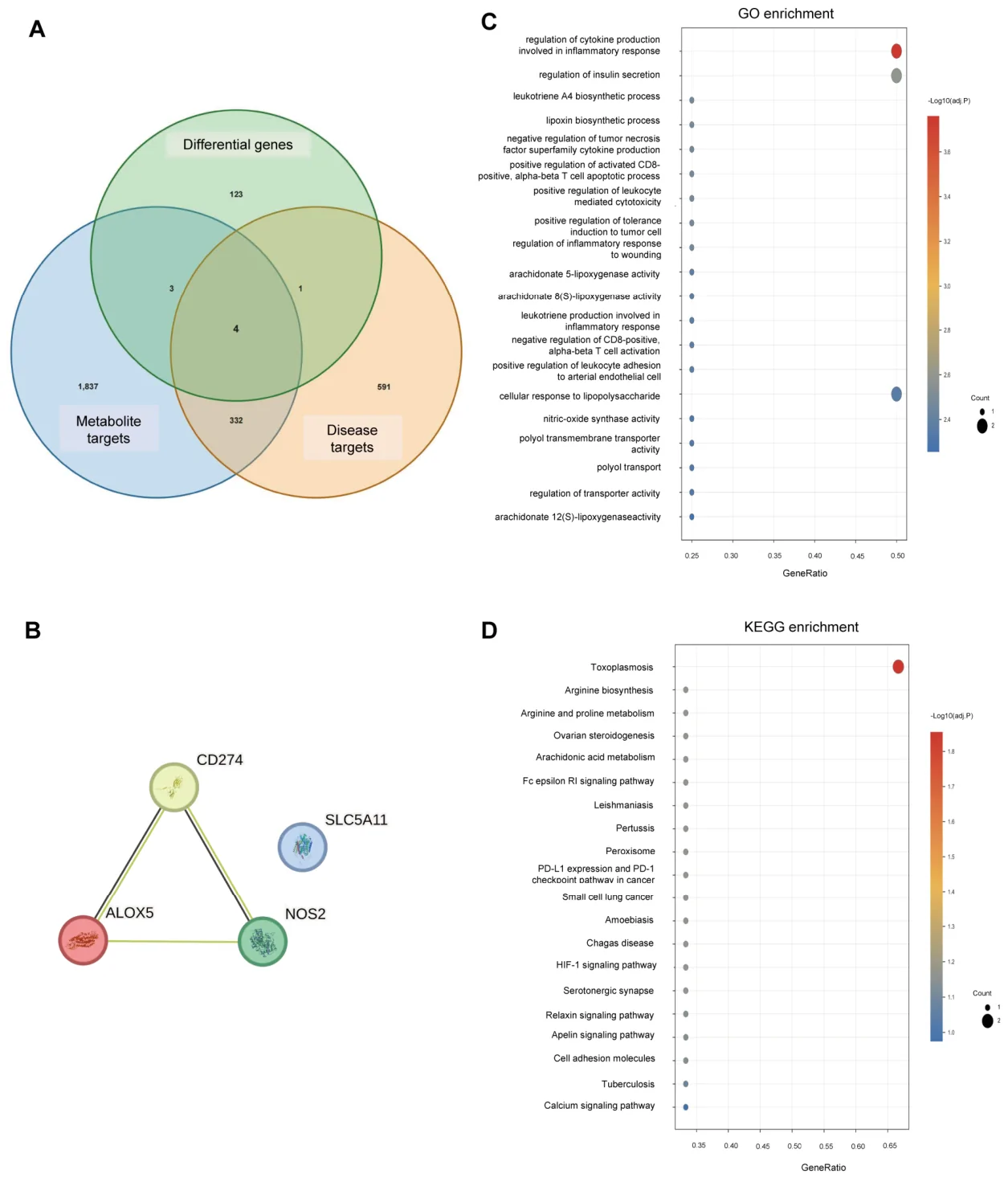
Target prediction and enrichment analysis. (**A**) Venn diagram showing the intersection among diseases, metabolites, and differentially expressed genes. (**B**) Protein–protein interaction (PPI) network diagram. (**C**) GO enrichment analysis. (**D**) KEGG pathway enrichment analysis.

**Figure 6:**
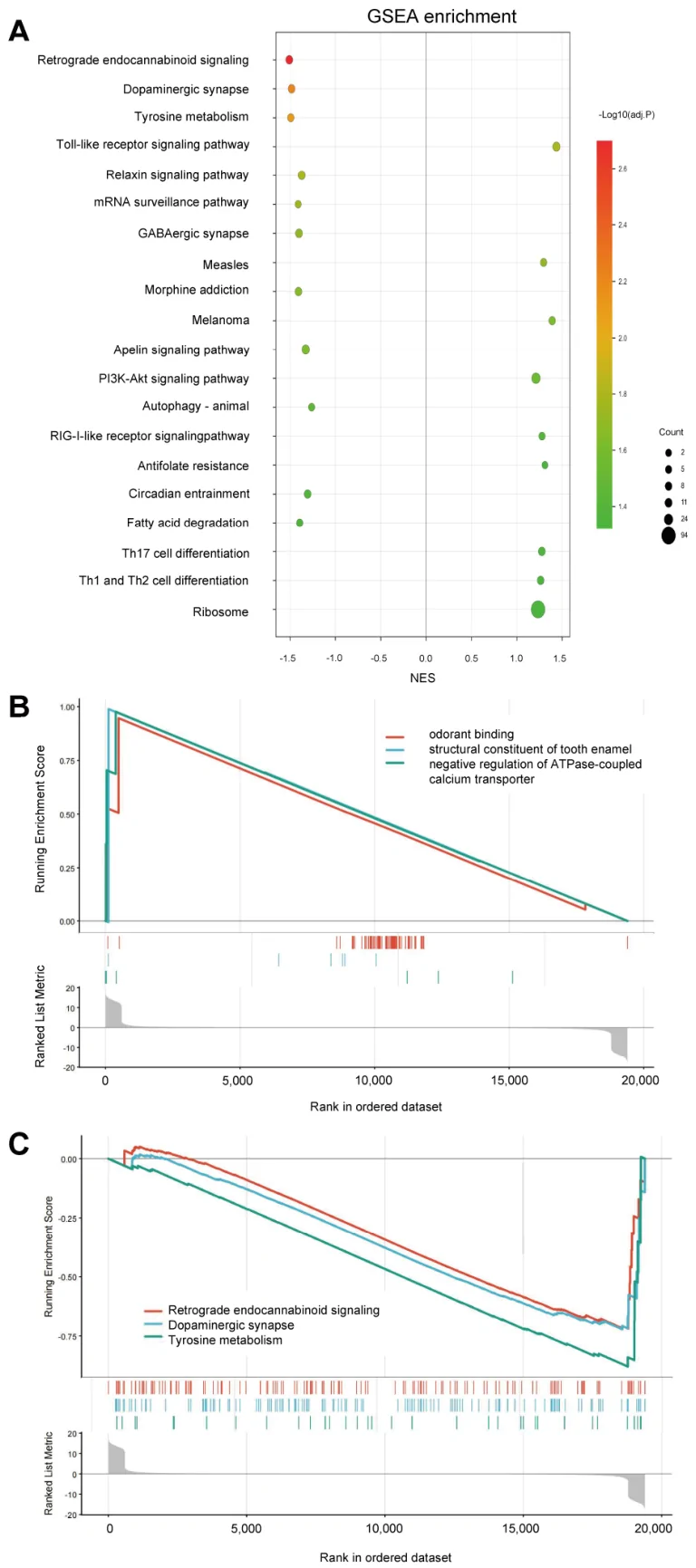
KEGG-GSEA enrichment and representative GSEA curves. (**A**) GSEA enrichment analysis. (**B**) GSEA enrichment plots of the top 3 significant GO terms. (**C**) GSEA enrichment plots of the top 3 significant KEGG pathways.

**Figure 7:**
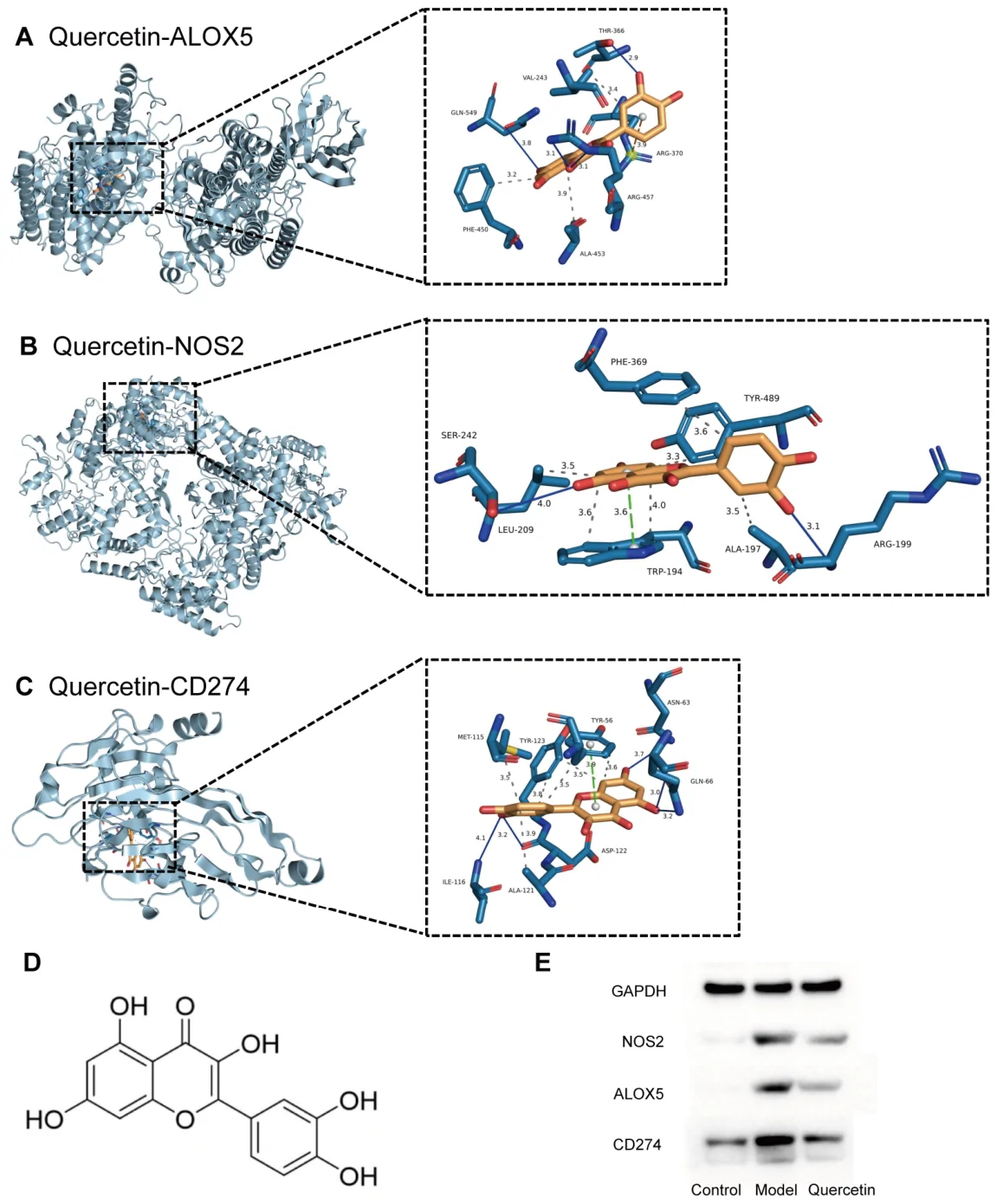
Molecular interaction and Western blot analysis. (**A**–**C**) Overall structure of the receptor and magnified view of the binding pocket. (**D**) Chemical structure of quercetin. (**E**) Western blot analysis showing the detected targets and treatment groups.

**Figure 8:**
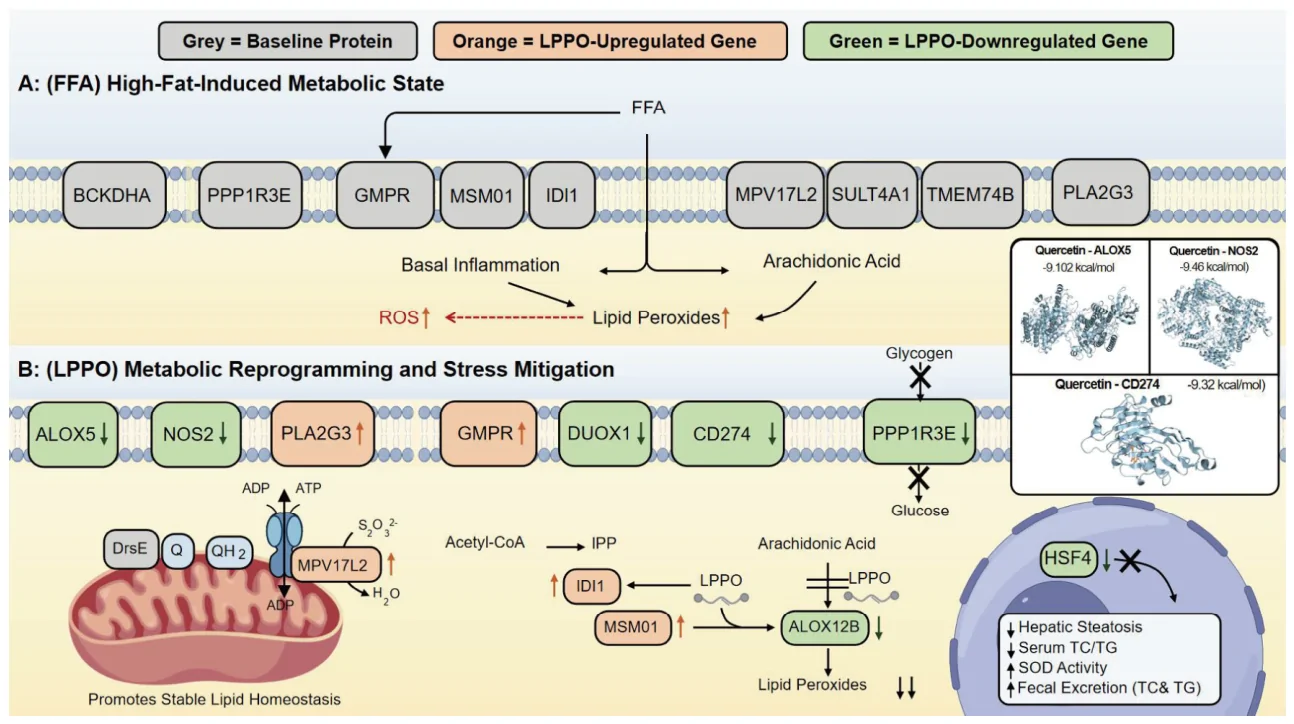
Proposed mechanism of LPPO in metabolic reprogramming and lipid utilization. (**A**) Schematic of high-fat induced state with basal inflammation and lipid peroxides. (**B**) LPPO-mediated reprogramming via target genes and mitochondrial membrane protection. Upward (↑) and downward (↓) arrows alongside genes or parameters indicate upregulation/increase and downregulation/decrease, respectively; solid and dashed connecting arrows represent metabolic or signaling pathways, while crossed arrows indicate pathway inhibition. Created in BioRender. Xiao, L. (2026). https://BioRender.com/80m7oed.

**Table 1:** Polycyclic Phenolic Compounds Identified by UPLC-MS/MS.

RT [min]	*m*/*z*	Name	Reference Ion	Formula	Relative Abundance		
					1	2	3
3.45	291.09	(-)-Epicatechin	[M+H]^+^	C_15_H_14_O_6_	9,321,954,654	7,755,535,810	8,687,496,693
3.72	577.13	Procyanidin A2	[M+H]^+^	C_30_H_24_O_12_	3,681,049,003	3,071,675,143	3,458,092,591
3.58	611.16	Rutin	[M+H-H2O]^+^	C_27_H_30_O_16_	1,328,188,921	1,816,507,908	1,463,320,384
3.66	303.05	Quercetin	[M+H]^+^	C_15_H_10_O_7_	253,629,340	391,143,104	268,543,802
3.87	275.09	Phloretin	[M+H]^+^	C_15_H_14_O_5_	283,992,696	300,918,593	281,483,803
0.70	307.08	Epigallocatechin	[M+H]^+^	C_15_H_14_O_7_	110,220,231	135,001,120	150,301,951
3.21	449.11	Cyanidin-3-O-galactoside	[M]^+^	C_21_H_21_O_11_	100,342,078	86,315,471	179,660,183
1.82	389.04	Dracorhodin perchlorate	[M+Na]^+^	C_17_H_15_ClO_7_	41,320,688	174,889,152	145,745,730
3.45	139.04	Afzelechin	[M+H]^+^	C_15_H_14_O_5_	128,895,402	108,994,018	121,541,031
0.63	273.08	Naringenin	[M+H]^+^	C_15_H_12_O_5_	108,323,719	105,591,768	97,674,914
4.20	287.06	Fisetin	[M+H]^+^	C_15_H_10_O_6_	20,674,911	116,174,117	40,303,338
3.44	273.08	L-(-)-Catechin	[M+Na]^+^	C_15_H_14_O_6_	53,662,626	45,183,086	52,462,730
0.69	335.04	Caftaric acid	[M+Na]^+^	C_13_H_12_O_9_	45,211,044	37,372,310	45,976,726
3.41	579.15	Procyanidin B3	[M+Na]^+^	C_30_H_26_O_12_	24,464,644	45,749,365	22,681,664
3.87	437.14	Phlorhizin	[M+H]^+^	C_21_H_24_O_10_	25,755,335	27,519,680	25,406,250
3.70	317.07	Isorhamnetin	[M+Na]^+^	C_16_H_12_O_7_	15,742,802	22,613,575	17,015,530
3.68	593.15	Kaempferol-3-O-rutinoside	[M-H]^−^	C_27_H_30_O_15_	1,135,440,724	1,667,793,445	1,166,008,118
3.45	335.08	Catechin	[M-H]^−^	C_15_H_14_O_6_	1,065,356,339	928,713,638	1,040,493,983
3.62	577.13	Procyanidin B1	[M-H]^−^	C_30_H_26_O_12_	435,942,144	409,261,302	411,205,536
7.40	192.93	Isoferulic acid	[M-H]^−^	C_10_H_10_O_4_	173,511,333	175,156,232	187,934,562
3.69	623.16	Isorhamnetin-3-rutinoside	[M-H]^−^	C_28_H_32_O_16_	106,468,328	162,664,610	117,296,856
3.34	865.19	Procyanidin C1	[M-H]^−^	C_45_H_38_O_18_	53,825,829	44,151,978	44,647,326

**Table 2:** Active Ingredient Targets.

Polyphenol Metabolites	Metabolite Targets
Quercetin	431
Catechin	215
Isorhamnetin	172
Rutin	122
(-)-Epicatechin	118

**Table 3:** Binding energy table.

Receptor	Ligand	Binding Energy (kcal/mol)
ALOX5	Quercetin	−9.102
NOS2	Quercetin	−9.46
CD274	Quercetin	−9.32

## Data Availability

The original contributions presented in the study are included in the article and Supplementary Materials; further inquiries can be directed to the corresponding author.

## Supplementary Materials

The following supporting information can be downloaded at: https://www.mdpi.com/article/10.3390/antiox15101246/s1, Figure S1: UPLC-MS/MS total ion chromatograms (TIC) of LPPO; Figure S2: Transcriptomic landscape and functional enrichment analysis across different experimental groups; Figure S3: General physiological parameters of mice over an 8-week period; Table S1: Quantitative evaluation of NAFLD Activity Score (NAS) based on H&E staining.

## Abbreviations

The following abbreviations are used in this manuscript:

5-HETE	5-hydroxyeicosatetraenoic acid
AGC	Automatic gain control
ALOX12B	Arachidonate 12-lipoxygenase, 12R type
ALOX5	Arachidonate 5-lipoxygenase
AMPK	Adenosine 5′-monophosphate-activated protein kinase
ANOVA	Analysis of variance
BCA	Bicinchoninic acid
BCKDHA	Branched-chain keto acid dehydrogenase E1 subunit alpha
CCK-8	Cell Counting Kit-8
CD274	Cluster of differentiation 274 (PD-L1)
cDNA	Complementary deoxyribonucleic acid
CTD	Comparative Toxicogenomics Database
DDA	Data-dependent acquisition
DEG	Differentially expressed gene
DMEM	Dulbecco’s modified Eagle’s medium
DUOX1	Dual oxidase 1
ENC1	Ectodermal-neural cortex 1
FBS	Fetal bovine serum
FC	Fold change
FDR	False discovery rate
FFA	Free fatty acid
GAPDH	Glyceraldehyde-3-phosphate dehydrogenase
GMPR	Guanosine monophosphate reductase
GO	Gene Ontology
GSEA	Gene Set Enrichment Analysis
H&E/HE	Hematoxylin and eosin
HCD	Higher-energy collisional dissociation
HDL-C	High-density lipoprotein cholesterol
HESI	Heated electrospray ionization
HFD	High-fat diet
HIF-1α	Hypoxia-inducible factor 1-alpha
HMDB	Human Metabolome Database
IDI1	Isopentenyl-diphosphate delta-isomerase 1
KEGG	Kyoto Encyclopedia of Genes and Genomes
LDL-C	Low-density lipoprotein cholesterol
LPPO	Litchi pericarp oligomeric polyphenols
LTB4	Leukotriene B4
MASLD	Metabolic dysfunction-associated steatotic liver disease
MC	Model control
MDA	Malondialdehyde
MoNA	MassBank of North America
MPV17L2	MPV17 mitochondrial inner membrane protein-like 2
MS/MS	Tandem mass spectrometry
MSMO1	Methylsterol monooxygenase 1
NAFLD	Non-alcoholic fatty liver disease
NES	Normalized enrichment score
NF-κB	Nuclear factor kappa B
NIST	National Institute of Standards and Technology
NO	Nitric oxide
NOS2	Nitric oxide synthase 2 (inducible nitric oxide synthase)
Nrf2	Nuclear factor erythroid 2-related factor 2
OMIM	Online Mendelian Inheritance in Man
PCR	Polymerase chain reaction
PDB	Protein Data Bank
PDE6G	Phosphodiesterase 6G
PD-L1	Programmed death-ligand 1
PFA	Paraformaldehyde
PGC-1α	Peroxisome proliferator-activated receptor gamma coactivator 1-alpha
PLA2G3	Phospholipase A2 group III
PPI	Protein–protein interaction
PPP1R3E	Protein phosphatase 1 regulatory subunit 3E
PVDF	Polyvinylidene fluoride
qPCR	Quantitative real-time polymerase chain reaction
ROS	Reactive oxygen species
SD	Standard deviation
SDS-PAGE	Sodium dodecyl sulfate–polyacrylamide gel electrophoresis
SIRT1	Sirtuin 1
SLC5A11	Solute carrier family 5 member 11
SMILES	Simplified molecular-input line-entry system
SOD	Superoxide dismutase
SREBP-1c	Sterol regulatory element-binding transcription factor 1c
TBA	Total bile acids
TC	Total cholesterol
TCMSP	Traditional Chinese Medicine Systems Pharmacology
TG	Triglycerides
TLR4	Toll-like receptor 4
UPLC-MS/MS	Ultra-performance liquid chromatography–tandem mass spectrometry
