# Supplementary material for: Integrated Multi-Omics and Network Pharmacology Reveal the Targeted Alleviation of MASLD by Litchi Pericarp Oligomeric Polyphenols via Quercetin
Source: Antioxidants (Basel). 2026 Sep 27;15(10):1246. doi: 10.3390/antiox15101246 (PMC13700870; doi:10.3390/antiox15101246)
Supplement: Supplementary file 1 [file antioxidants-15-01246-s001.zip › antioxidants-4558049-supplementary.pdf]

Supplementary Material

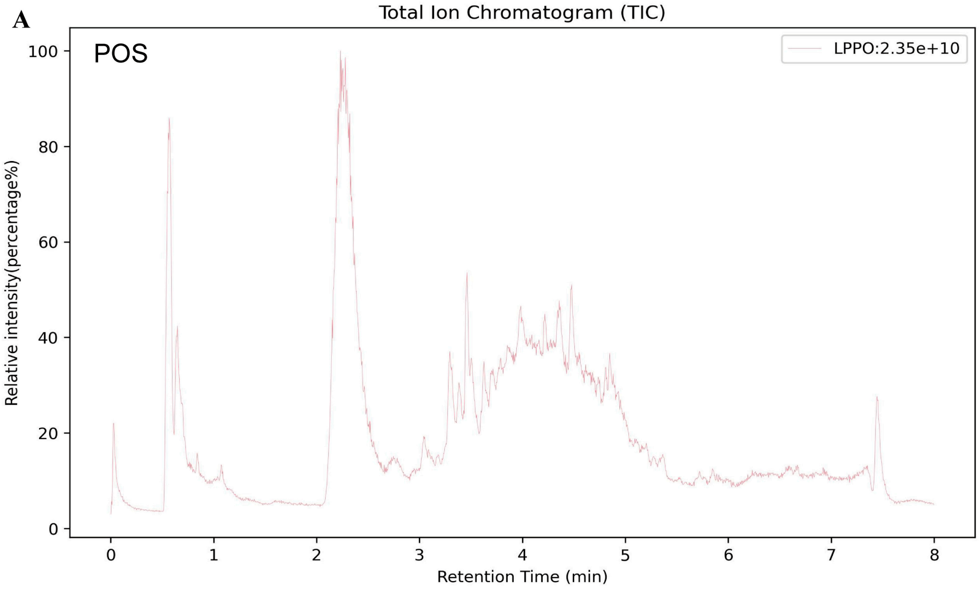

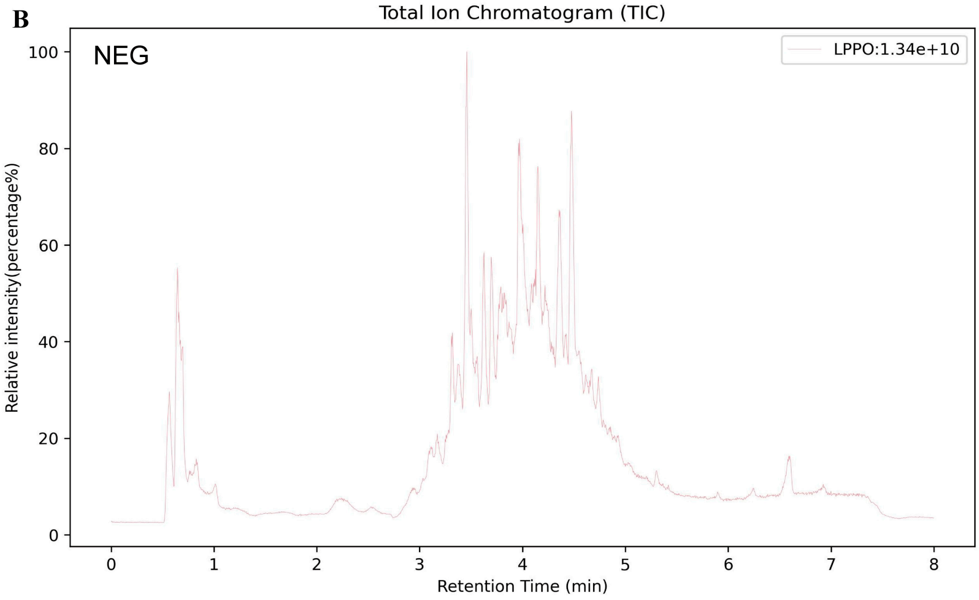

Supplementary Figure S1. UPLC-MS/MS total ion chromatograms (TIC) of LPPO. (A) Total ion chromatogram in positive ionization mode (POS); (B) Total ion chromatogram in negative ionization mode (NEG). The TIC profiles provide a comprehensive chemical fingerprint of the active components in LPPO.

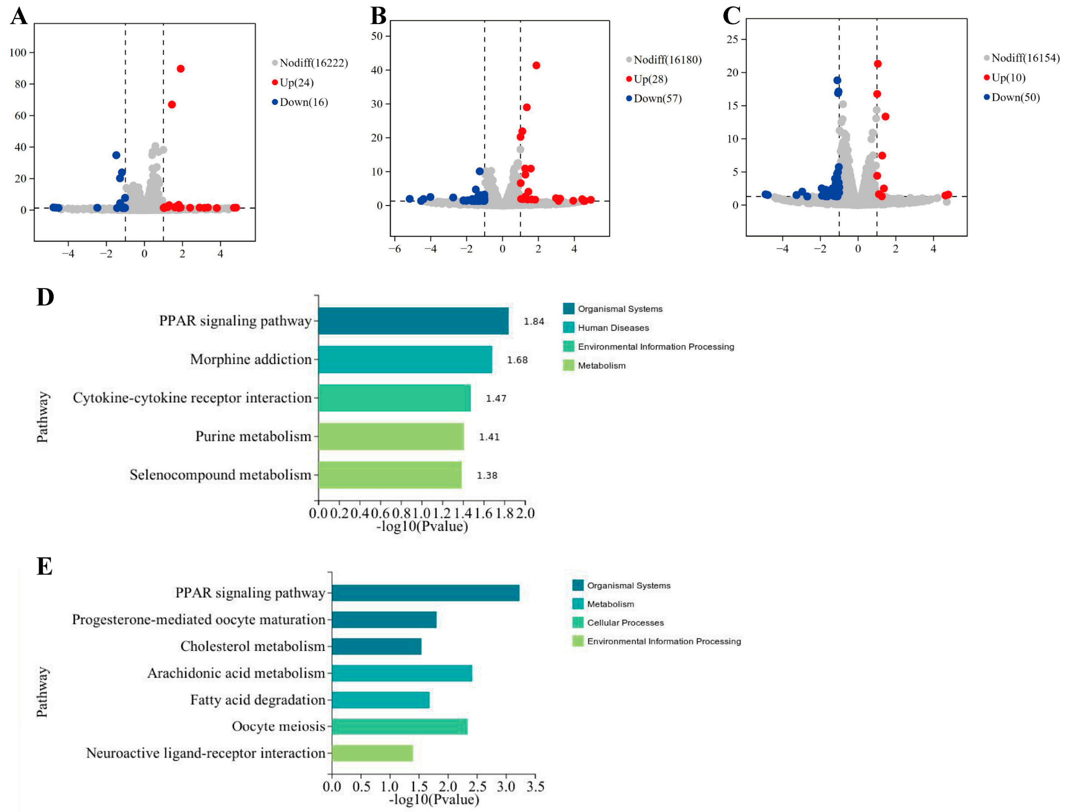

Supplementary Figure S2. Transcriptomic landscape and functional enrichment analysis across different experimental groups. (A–C) Volcano plots illustrating the differentially expressed genes (DEGs) in (A) NC vs. MC, (B) NC vs. HD, and (C) MC vs. HD comparisons. Red and blue dots represent significantly up-regulated and down-regulated genes, respectively ($|\log_2 FC| > 1$ and $p < 0.05$). (D–E) KEGG pathway enrichment analysis showing the primary biological pathways altered in (D) NC vs. MC and (E) NC vs. HD groups, indicating significant metabolic reprogramming.

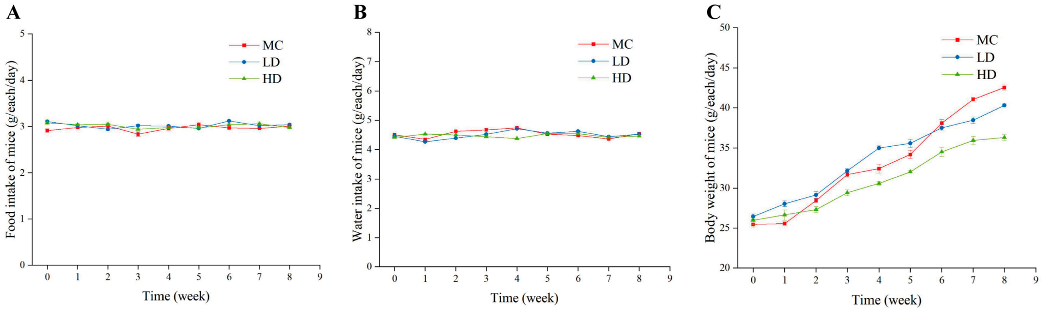

Supplementary Figure S3. General physiological parameters of mice over an 8-week period. (A) Food intake of mice; (B) Water intake of mice; (C) Body weight of mice.

Supplementary Table S1. Quantitative evaluation of NAFLD Activity Score (NAS) based on H&E staining

Experimental Group	Hepatic Steatosis (0–3)	Lobular Inflammation (0–3)	Hepatocellular Ballooning (0–2)	Total NAS Score (0–8)
MC	3 ± 0.00^{a}	2 ± 0.50^{a}	2 ± 0.50^{a}	7 ± 0.71^{a}
LD	1 ± 0.5^{b}	1 ± 0.50^{b}	1 ± 0.00^{b}	3 ± 0.71^{b}
HD	0 ± 0.00^{c}	0 ± 0.00^{c}	0 ± 0.00^{c}	0 ± 0.00^{c}
